# Phototrophic extracellular electron uptake is linked to carbon dioxide fixation in the bacterium *Rhodopseudomonas palustris*

**DOI:** 10.1038/s41467-019-09377-6

**Published:** 2019-03-22

**Authors:** Michael S. Guzman, Karthikeyan Rengasamy, Michael M. Binkley, Clive Jones, Tahina Onina Ranaivoarisoa, Rajesh Singh, David A. Fike, J. Mark Meacham, Arpita Bose

**Affiliations:** 1 Department of Biology, Washington University in St. Louis, St. Louis, MO 63130 USA; 2 Department of Mechanical Engineering and Materials Science, Washington University in St. Louis, St. Louis, MO 63130 USA; 3 Department of Earth and Planetary Sciences, Washington University in St. Louis, St. Louis, MO 63130 USA; 4 Institute of Materials Science Engineering, Washington University in St. Louis, St. Louis, MO 63130 USA

## Abstract

Extracellular electron uptake (EEU) is the ability of microbes to take up electrons from solid-phase conductive substances such as metal oxides. EEU is performed by prevalent phototrophic bacterial genera, but the electron transfer pathways and the physiological electron sinks are poorly understood. Here we show that electrons enter the photosynthetic electron transport chain during EEU in the phototrophic bacterium *Rhodopseudomonas palustris* TIE-1. Cathodic electron flow is also correlated with a highly reducing intracellular redox environment. We show that reducing equivalents are used for carbon dioxide (CO_2_) fixation, which is the primary electron sink. Deletion of the genes encoding *ruBisCO* (the CO_2_-fixing enzyme of the Calvin-Benson-Bassham cycle) leads to a 90% reduction in EEU. This work shows that phototrophs can directly use solid-phase conductive substances for electron transfer, energy transduction, and CO_2_ fixation.

## Abstract (web summary)

Extracellular electron uptake (EEU) is the ability of microbes to take up electrons from solid-phase conductive substances such as metal oxides. Here, Guzman et al. show that electrons enter the photosynthetic electron transport chain and are used for CO_2_ fixation during EEU in a phototrophic bacterium.

## Introduction

Microbial phototrophic carbon dioxide (CO_2_) fixation accounts for substantial primary productivity on Earth^1^. Anoxygenic phototrophs, which include the green and purple sulfur bacteria, are metabolically versatile microbes that oxidize an array of inorganic compounds^2^. These include H_2_S, H_2_, Fe^2+^, and intriguingly, solid-phase conductive substances (SPCSs) via a process called extracellular electron uptake (EEU)^3–5^. Microbial oxidation–reduction reactions with SPCSs play an important role in soil, marine sediments, and deep subsurface microbial communities^6^. The cellular electron transfer and metabolic pathways that allow photoautotrophs to utilize SPSCs via EEU, however, are largely unknown. It remains elusive whether electron uptake from SPSCs is connected to cyclic photosynthetic electron transfer and/or the generation of reducing equivalents for CO_2_ fixation. Subsequently, the ecological and evolutionary role of phototrophic EEU remains poorly understood.

Poised electrodes in bioelectrochemical systems (BESs) have been used as proxies of microbial interactions with natural SPCSs, such as metal oxides^5,7^. Studies using BESs have led to fundamental insights into the molecular underpinnings of extracellular electron transfer in mineral respiring microbes^4,8^. These studies have revealed extracellular electron transfer is a widespread process in nature^4,5,8^. Furthermore, microbe-electrode interactions have been leveraged for biotechnological applications such as microbial electrosynthesis^9^. Our laboratory^3,10^, and others^11,12^, have recently applied BESs to better understand the molecular details of microbial phototrophic EEU. This has led to the discovery of at least two pure cultures capable of EEU from electrodes, the anoxygenic phototrophs *Rhodopseudomonas palustris* TIE-1^3^ and *Prosthecochloris aestuarii*^12^. Thus far, only *R. palustris* TIE-1 is genetically tractable^13^, and as such is a model system for studying EEU.

Here, we use an interdisciplinary approach combining bioelectrochemistry, molecular biology, isotope-based geochemistry, nanotechnology, and microfluidics, to examine the bioenergetic pathways and physiological electron sinks that allow photoautotrophs to use SPCSs as electron donors. Using TIE-1 as a model system we show that EEU is linked to the photosynthetic electron transport chain (pETC), and that this process leads to cells becoming highly reduced with respect to both the intracellular nicotinamide adenine dinucleotide [NAD(H)] and nicotinamide adenine dinucleotide phosphate [NAD(P)(H)] pools. We also test the ability of TIE-1 to fix CO_2_ during EEU using labeling studies. These data show that EEU results in CO_2_ fixation to biomass via the Calvin-Benson-Bassham (CBB) cycle. Furthermore, using mutant analysis we observe that the CBB cycle is the primary electron sink. Overall, our results trace the path of electrons following EEU through the electron transport chain and cellular metabolism.

## Results

### EEU is linked to photosynthetic electron transfer

EEU from metal oxides or poised electrodes into bacterial cells has been observed in pure cultures^3–6,12,14–20^, and mixed microbial communities^4,5,21–23^. However, the electron transfer pathways that underlie EEU have only been probed in chemotrophic microbes^14,15,18,24^. In phototrophic microbes, it is unknown if electrons from a cathode enter the pETC and if this activity is important for the establishment of a proton motive force (PMF), ATP synthesis, or the generation of reducing equivalents. Bioelectrochemical studies traditionally rely upon macroscale (>500 mL) or mesoscale (0.2–500 mL) BESs that are scaled for biomass production^25^. In such BESs it is difficult to isolate the response of surface-attached cells. This is because other factors like the influence of planktonic cells^3,10^, extracellular enzymes^26^, and abiotic reactions confound the interpretation of electrochemical data^3,10^. Being able to collect electrochemical data from surface-attached cells exclusively would shed light on whether EEU leads to electron transfer into the pETC.

**Fig. 1:**
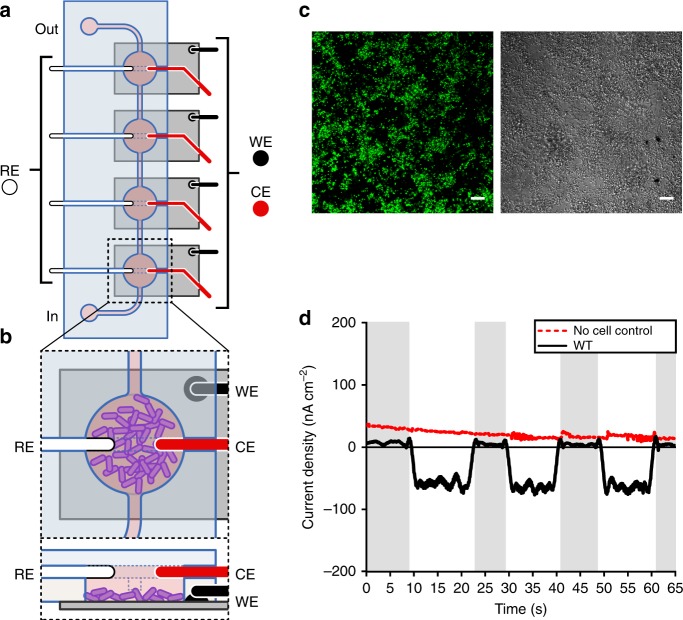
Extracellular electron uptake in the micro-bioelectrochemical cell. **a** Schematic drawing of a single, four-chamber micro-bioelectrochemical (µ-BEC) with **b** microbial cells attached to the indium tin oxide (ITO) working electrode (WE). The reference (RE) and counter (CE) electrodes are silver and platinum wires, respectively (not drawn to scale). **c** Confocal micrograph of *Rhodopseudomonas palustris* TIE-1 biofilms attached to the WE under poised conditions using LIVE/DEAD® staining. Green cells are viable. Scale bars are 10 µm. **d** Current densities for TIE-1 wild-type (WT) (black) in the µ-BEC under illuminated and dark conditions (shaded regions) compared to a ‘No cell control’ reactor (red). Data shown are representative of three experiments. Source data are provided as a Source Data File

To achieve this, we designed and constructed a microfluidic bioelectrochemical cell (µ-BEC) (Fig. 1a, Supplementary Figure 1). The µ-BEC is a four-chamber, three-electrode, small-volume (1.6 µL per well) BES that is compatible with confocal microscopy (Fig. 1a) (see Methods for a complete description of the µ-BEC design and assembly). Its major advantage is that it allows us to study surface-attached cells exclusively as planktonic cells can be washed out with microfluidic control (Fig. 1b). Appropriately grown microbial cells were incubated in µ-BECs for ~120 h at +100 mV vs. standard hydrogen electrode (SHE) under continuous illumination. Once we obtained stable current densities under illuminated conditions (~ −100 nA cm^−2^), planktonic cells were washed out of the system with microfluidic control. Medium flow was turned off following this wash because constant flow led to excessive noise in the electrochemical data. To determine that we only had surface-attached cells and no plankton, we performed confocal fluorescence microscopy with LIVE/DEAD® staining in the intact µ-BEC. We observed surface-attached cells in single-layer biofilms (Fig. 1c and Supplementary Figure 2a). Previous studies have shown that EEU-capable microbes, including TIE-1, make single-layer biofilms on electrodes^3,9,27–29^. Furthermore, we were unable to detect the presence of any motile planktonic cells in the µ-BEC.

We used the above approach to obtain surface-attached cells in the µ-BEC and used these biofilms to study the influence of light and chemical inhibitors on EEU. Confocal imaging using LIVE/DEAD® staining was performed in the intact µ-BEC after these tests that typically lasted for a few minutes (see Methods for details). We observed light-stimulated EEU by pre-established wild-type (WT) TIE-1 biofilms (Fig. 1d). Upon illumination, biofilms reached stable current densities within ~1–2 s and typically reached a maximum of ~ −100 nA cm^−2^ (Supplementary Table 1,2,3). Overall, the µ-BEC replicates the biofilm architecture reported in bulkier systems and permits reproducible measurements of EEU by surface-attached cells.

**Fig. 2:**
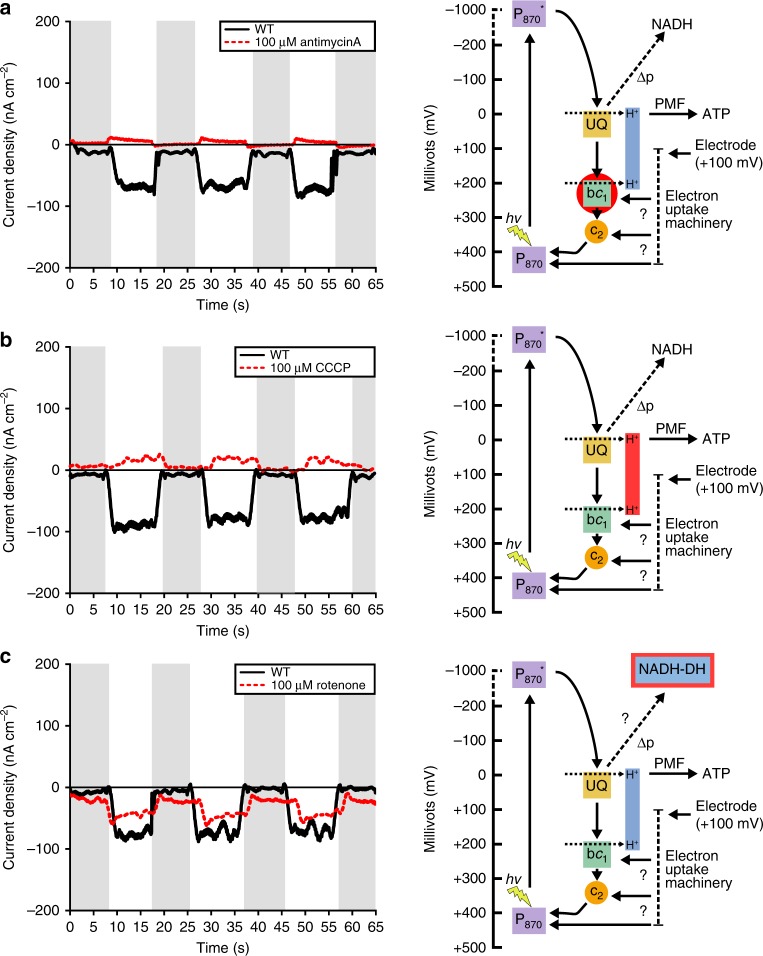
Photosynthetic electron transfer is required for extracellular electron uptake. Current densities of TIE-1 wild-type (WT) in response to inhibition of the photosynthetic ETC under illuminated and dark (shaded regions) conditions with (**a**) antimycin A, (**b**) carbonyl cyanide *m*-chlorophenyl hydrazine (CCCP), and (**c**) rotenone. Data shown are representative of three experiments. Each current density diagram (left) is followed by the proposed path of electron flow (right). The site of chemical inhibition is indicated by a red halo on the electron path diagrams. P_870_ (photosystem), P_870_^*^ (excited photosystem), UQ (ubiquinone), *bc*_1_ (cytochrome *bc*_1_), *c*_2_ (cytochrome *c*_2_), NADH-DH (NADH dehydrogenase), Δp (proton gradient), H^+^ (protons), *hv* (light), ? (currently unknown), PMF (proton motive force) and ATP (adenosine triphosphate). Source data are provided as a Source Data File

To better understand electron flow during EEU we pursued a chemical probe-based approach to selectively inhibit key proteins involved in cyclic pETC. TIE-1 and related anoxygenic phototrophs use cyclic photosynthesis^30^ to generate energy (Fig. 2). The photosystem (P_870_) is reported to be at the potential of +450 mV^30^. Quinones reduced by the photosynthetic reaction center (P_870_*) donate electrons to the proton-translocating cytochrome *bc*_1_^31^. Electrons are then transferred to cytochrome *c*_2_, and cycled back to the reaction center^30^. To test whether cytochrome *bc*_1_ is involved in EEU, we used antimycin A, a specific inhibitor of cytochrome *bc*_1_^32^ to block cyclic pETC (Fig. 2a). Antimycin A is a quinone analog that blocks the Q_i_ site of cytochrome *bc*_1_, inhibiting electron transfer from ubiquinol to cytochrome *b*, thus disrupting the proton motive Q cycle^31,32^. We observed a decrease in current uptake with antimycin A treatment (Fig. 2a, Supplementary Table 1). Current density became anodic (positive current) under phototrophic conditions (12.46 ± 1.34 nA cm^−2^; *P* < 0.0001, one-way ANOVA) relative to untreated controls (−85.5 ± 5.42 nA cm^−2^) but reverted to cathodic (negative current) densities under dark conditions (−3.46 ± 1.80 nA cm^−2^; *P* = 0.0006, one-way ANOVA) (Fig. 2a). Importantly, we did not observe a difference in the number of live/dead cells attached to electrodes in inhibitor treated vs. untreated control reactors (Supplementary Figure 2). These data suggest that electrons enter the pETC and that cytochrome *bc*_1_ is involved in electron flow during EEU.

Cyclic electron flow by the pETC is important for the establishment of a PMF that drives ATP production^30^. To investigate whether a proton gradient is important for EEU, we exposed TIE-1 biofilms to the protonophore carbonyl cyanide *m*-chlorophenyl hydrazone (CCCP) (Fig. 2b). CCCP is a lipid-soluble molecule that dissipates the PMF such that electron transfer is uncoupled from ATP synthesis^30,33^. We observed a decrease in current uptake heading toward anodic current under illuminated conditions upon CCCP treatment (21.2 ± 9.13 nA cm^−2^; *P* < 0.0001, one-way ANOVA) compared to untreated controls (−113.5 ± 21.7 nA cm^−2^) (Fig. 2b, Supplementary Table 2). Current uptake was not different between CCCP (−18.4 ± 14.0 nA cm^−2^; *P* = 0.8666, one-way ANOVA) and untreated controls (−17.52 ± 3.41 nA cm^−2^) under dark conditions (Fig. 2b). These results demonstrate that a PMF is required for EEU. Furthermore, dark EEU is not PMF-dependent as EEU can occur in the presence of CCCP.

The proton-translocating NADH dehydrogenase oxidizes NADH to generate a PMF for ATP production^30^. NADH dehydrogenase can also function in reverse to catalyze uphill electron transport from the ubiquinone pool to reduce NAD^+^ in the anoxygenic phototrophs *Rhodobacter capsulatus*^34^ and *R. sphaeroide*s^35^. Its activity is linked to redox homeostasis and carbon metabolism in these organisms^36^. To investigate whether NADH dehydrogenase has a role in EEU in TIE-1, we treated cells with the NADH dehydrogenase inhibitor rotenone^37^. Rotenone blocks electron transfer from the iron-sulfur clusters in NADH dehydrogenase to ubiquinone^38^ (Fig. 2c). In illuminated biofilms, we observed a ~20% decrease in current uptake with low rotenone concentrations (25 µM; −71.8 ± 2.02 nA cm^−2^; *P* < 0.0001, one-way ANOVA) compared to untreated controls (−94.7 ± 3.61 nA cm^−2^), and up to a ~50% decrease with exposure to high rotenone concentrations (100 µM; −41.6 ± 4.55 nA cm^−2^; *P* < 0.0001, one-way ANOVA) (Fig. 2c, Supplementary Table 3). The current uptake maxima were markedly lower under these conditions (Supplementary Table 3). After initial current uptake, we observed that rotenone-treated cells showed lowered current uptake post light exposure (Fig. 2c). It is unclear if this reduction is solely due to lowered current uptake or a combination of both lowered current uptake and increased electron donation to the electrode. The reduction in current uptake could also be a consequence of overreduction of the ubiquinone pool as has been observed in *R. sphaeroides* NADH dehydrogenase mutants^38,39^. Because we observe only a partial lowering of current uptake with NADH dehydrogenase inhibition (Fig. 2c), the cell likely has additional sinks for using reduced ubiquinone.

CCCP and antimycin A treatment both resulted in anodic current generation under illuminated conditions. Although the magnitude of the electrochemical response was different in the two cases, these data suggest that when the pETC is inhibited, TIE-1 cells likely transfer electrons to the poised electrodes by using them as an electron sink. Overall, our inhibitor studies show that (1) electrons enter the pETC of TIE-1 following EEU; (2) PMF is required for light-dependent EEU; (3) cytochrome *bc*_1_ is involved in electron flow; and that (4) NADH dehydrogenase plays an important role in EEU.

### EEU leads to an imbalance in intracellular redox

**Fig. 3:**
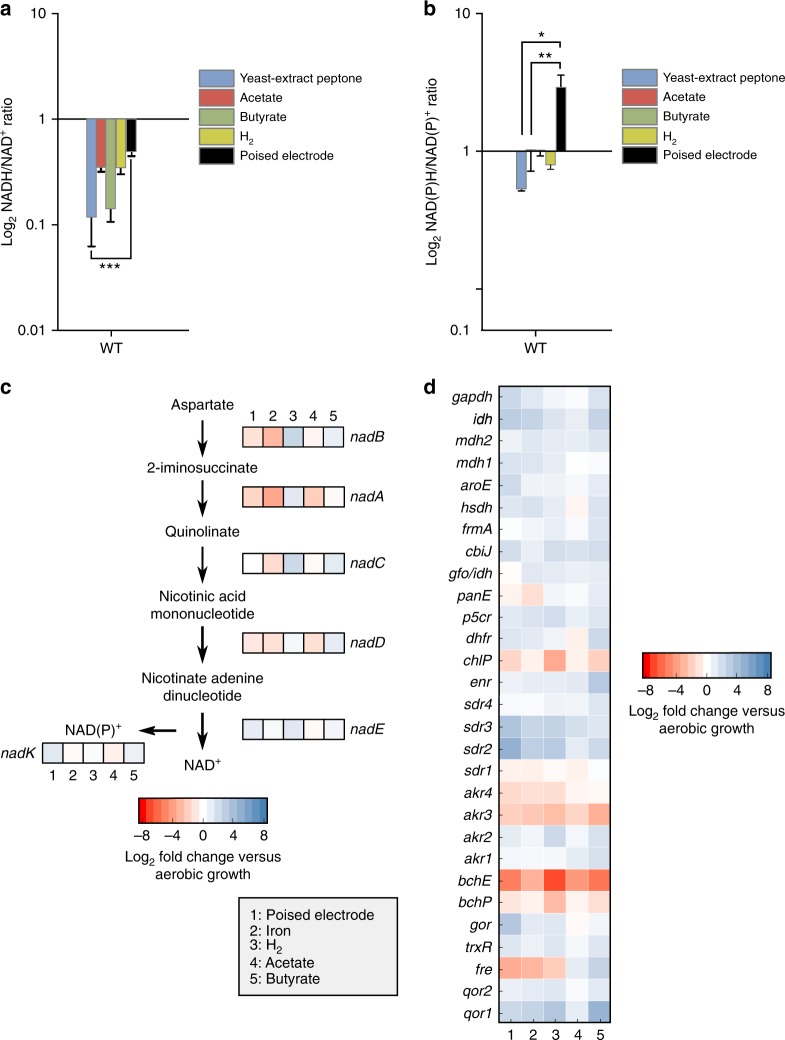
Extracellular electron uptake leads to a reducing intracellular redox environment. **a** TIE-1 WT NADH/NAD^+^ and **b** NAD(P)H/NAD(P)^+^ ratios under various growth conditions. Conditions tested: yeast-extract peptone (blue); photoheterotrophy with acetate (red) and butyrate (green); and photoautotrophy with H_2_ (yellow) or a poised electrode (black). Data are means ± s.e.m. of three biological replicates assayed in triplicate. The *P* values were determined by one-way ANOVA followed by a pairwise test with Bonferroni adjustment (*P < 0.05, **P < 0.01, ***P < 0.0001; ns, not significant). **c** Transcriptomic analysis of the de novo NAD biosynthesis pathway under various photoautotrophic and photoheterotrophic growth conditions. **d** Genome-wide transcriptomic analysis of NAD(P)^+^/H-requiring reactions. Source data (and reactions not mentioned in text) are provided as a Source Data File

NAD and its reduced state NADH are essential cofactors for microbes^30^. NADH can be converted to NAD(P)H via NAD(P)^+^ transhydrogenase^40^ (Rpal_4660-4662). NADH and NAD(P)H are key electron donors for biosynthetic reactions, including CO_2_ fixation. To better understand how the intracellular redox pool is affected by EEU, we examined the NADH/NAD^+^ and NAD(P)H/NAD(P)^+^ ratios in planktonic cells^41^. We compared these ratios to aerobic chemoheterotrophy (i.e., the inoculum) and phototrophic conditions where other electron donors were provided. We observed that the NADH/NAD^+^ ratio in the WT during EEU was higher than aerobic chemoheterotrophic growth (Fig. 3a). The NADH/NAD^+^ ratio was also higher than phototrophic growth on hydrogen (H_2_) or photoheterotrophic growth on acetate or butyrate (*P* < 0.0001; Fig. 3a, one-way ANOVA). The NAD(P)H/NAD(P)^+^ ratio was also highest during EEU compared to other conditions (*P* < 0.01, one-way ANOVA; Fig. 3b).

Analysis of intracellular redox suggests that EEU may lead to a highly-reduced environment in the cell. The lack of NAD^+^ or NAD(P)^+^ might require de novo NAD synthesis for cellular survival. Therefore, NAD biosynthesis might increase during EEU. We analyzed the expression of the de novo (aspartate-dependent) NAD biosynthesis pathway^42^ in the WT transcriptome encoded by *nadABCDE*. This pathway was not differentially expressed under any phototrophic condition, including EEU (Fig. 3c). NAD kinase which converts NAD^+^ to NAD(P)^+^ was also not differentially expressed under the conditions tested (Fig. 3c). These data suggest NAD biosynthesis does not increase at the level of gene expression during EEU despite a highly-reduced redox pool.

We reasoned that NAD(P)^+^ consuming and/or producing reactions might be upregulated during EEU to maintain redox balance. Therefore, we assessed the expression of NAD(P)^+^/H-requiring reactions across the TIE-1 genome. We observed that the majority of NAD(P)^+^/H-requiring reactions were downregulated under phototrophic conditions (Fig. 3d). Interestingly, an NADP-dependent FMN-binding flavin reductase-like protein (*fre*) was upregulated during photoautotrophic growth, increasing ~4-fold during EEU (Fig. 3d). A pair of NAD(P)^+^/H-dependent oxidoreductases (*akr3* and *akr4*) were also differentially expressed (Fig. 3d). *Akr3* was upregulated under all phototrophic conditions whereas *akr4* was specifically upregulated during phototrophic H_2_ oxidation and EEU. These data suggest that under EEU the cells are highly reduced and that the lack of oxidized NAD^+^ and/or NAD(P)^+^ is not relieved by de novo NAD biosynthesis. However, several NAD(P)^+^/H-dependent reactions are upregulated.

### EEU is linked to CO_2_ fixation via the CBB cycle

**Fig. 4:**
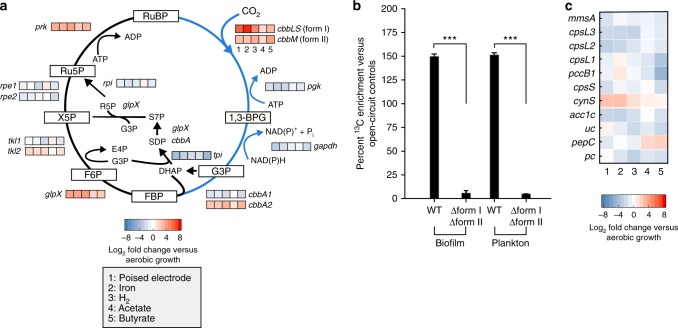
Extracellular electron uptake leads to carbon dioxide fixation. **a** Differential expression analysis of genes encoding Calvin-Benson-Bassham (CBB) cycle enzymes in TIE-1 wild-type (WT) under various photoautotrophic (poised electrodes, iron oxidation, and H_2_ oxidation) and photoheterotrophic growth conditions (acetate and butyrate). **b** ^13^CO_2_ incorporation under cathodic conditions in TIE-1 WT and the *ruBisCO* double mutant (∆form I ∆form II) biofilms and planktonic cells determined by secondary ion mass spectrometry (SIMS). Data are means ± s.e.m. of at least 25 cells. The *P* values were determined by one-way ANOVA followed by a pairwise test with Bonferroni adjustment (*P < 0.05, **P < 0.01, ***P < 0.0001; ns, not significant). **c** Differential expression analysis of CO_2_ and HCO_3_^−^ consuming reactions in TIE-1 WT. RuBP (Ribulose 1,5-bisphosphate), 1,3 BPG (1,3-bisphosphoglycerate), G3P (Glyceraldehyde 3-phosphate), FBP (Fructose 1,6-bisphosphate), F6P (Fructose 6-phosphate), X5P (Xylulose 5-phosphate), Ru5P (Ribulose 5-phosphate) and R5P (Ribose 5-phosphate). Source data (and reactions not mentioned in text) are provided as a Source Data File

Our data shows that EEU results in electron transfer to the pETC (Fig. 2), eventually producing NADH and NAD(P)H (Fig. 3). In anoxygenic phototrophs CO_2_ fixation is a major sink for NAD(P)H^30^. In our initial study on EEU by TIE-1, we observed that mRNA transcripts for genes encoding form I ribulose-1,5-bisphosphate carboxylase/oxygenase (RuBisCO) increased during EEU^3^. RuBisCO catalyzes CO_2_ fixation in many autotrophic organisms as part of the CBB cycle^30^. Therefore, we asked whether CO_2_ fixation occurs during EEU via RuBisCO. TIE-1 encodes two forms of RuBisCO: forms I (*cbbLS*) and II (*cbbM*)^43^. Using transcriptomic analysis, we analyzed the expression of the CBB cycle in TIE-1 and observed that form I *ruBisCO* was upregulated under all phototrophic conditions, but its expression was highest during EEU (~6-fold, *P* < 0.0001, one-way ANOVA) and phototrophic iron oxidation (~7-fold, *P* < 0.0001, one-way ANOVA) (Fig. 4a). Form II *ruBisCO* was expressed at similar levels across all phototrophic conditions (Fig. 4a). The other enzyme unique to the CBB cycle, phosphoribulokinase (Prk), was also upregulated during EEU (*P* < 0.0001, one-way ANOVA; Fig. 4a). Prk catalyzes the synthesis of the CO_2_ acceptor molecule, ribulose 1,5-bisphosphate (RuBP)^30^.

The expression of genes encoding CBB cycle-specific enzymes, including form I *ruBisCO*, suggests that CO_2_ fixation occurs during EEU. There are established methods for answering whether CO_2_ fixation is occurring in planktonic cells that can be grown in bulk^44,45^. However, in the case of EEU the cells attach to electrodes, which precludes us from using standard methodology. To overcome this, we employed secondary ion mass spectrometry (SIMS), and traced ^13^CO_2_ assimilation in TIE-1. The WT and a *ruBisCO* double mutant (∆*cbbLS* ∆*cbbM*) (Supplementary Table 4) were subjected to four treatments in BESs as follows: (1) poised electrodes with ^12^CO_2_; (2) poised electrodes with ^12^CO_2_ supplemented with 10% ^13^CO_2_ (poised + ^13^CO_2_); (3) electrodes at open circuit with ^12^CO_2_ (passing no current; control); and (4) electrodes at open circuit with ^12^CO_2_ supplemented with 10% ^13^CO_2_ (control + ^13^CO_2_) (Supplementary Figure 3). We chose to pre-grow cells under aerobic chemoheterotrophic conditions because the *ruBisCO* double mutant did not have a growth defect here compared to the WT (Supplementary Table 5). We used bulk BESs (~70 mL) here because they are closed systems, and do not lose CO_2_, unlike the μ-BEC, which is an anoxic microfluidic system under intermittent microfluidic flow.

Cells were cultivated for ~60 h, and planktonic and surface-attached cells (biofilms) were harvested for SIMS analysis. WT cells under poised conditions were enriched in ^13^C relative to the nonamended cells, indicating the assimilation of ^13^CO_2_ by both surface-attached and planktonic cells (Fig. 4b, Supplementary Table 6). The WT also increased in biomass above open circuit (Supplementary Figure 4). In contrast, the *ruBisCO* double mutant had a 96% reduction in ^13^CO_2_ assimilation compared to WT (Fig. 4b, Supplementary Table 7), a reduced capacity to take up electrons (Supplementary Figure 3) and no biomass increase (Supplementary Figure 4). These data demonstrate that EEU and CO_2_ assimilation are connected, and that RuBisCO catalyzes the major CO_2_ assimilation reaction in this system.

The planktonic and the surface-attached cells show the same level of ^13^C assimilation. This might be due to surface-attached cells and the plankton interacting dynamically with the electrode. To address this, we devised an experiment where pre-established biofilms (from 48 h bioreactor runs) on poised electrodes (biocathodes) were transferred into “plankton-free” bioreactors with fresh medium (Supplementary Figures 5). We observed that after 48 h current densities in “plankton-free” bioreactors were ~70% lower than the plankton-containing bioreactors (*P* < 0.05, one-way ANOVA; Supplementary Figure 5a–e). Plankton increased to nearly 0.06 OD_660_, while the biocathode remained fully colonized (Supplementary Figure 5a–c, f). In a reciprocal experiment, when new cell-free cathodes were installed in the plankton-containing bioreactors (used to obtain the biocathodes), current densities resembled the original levels (Supplementary Figure 5a–e). This suggests that the plankton retains the ability to attach to the electrodes after 48 h. These data, along with ^13^CO_2_ assimilation, suggests that planktonic cells in the bioreactors are interacting dynamically with the poised electrodes.

The uptake of ^13^CO_2_ in the *ruBisCO* double mutant (Fig. 4b) likely represents CO_2_ consuming reactions such as non-autotrophic carboxylases shown in Fig. 4c. Multiple carboxylases in the TIE-1 genome are expressed during EEU, however, many of these reactions are downregulated relative to chemoheterotrophic growth (Fig. 4c). *cynS*, which encodes cyanase is upregulated during EEU (*P* < 0.05, one-way ANOVA; Fig. 4c). Cyanase catalyzes the bicarbonate-dependent metabolism of cyanate, that accumulates as a byproduct of urea dissociation and/or carbamoyl phosphate decomposition^46^. Overall, our data suggest that RuBisCO is the primary reaction that is catalyzing CO_2_ fixation during EEU.

### The CBB cycle is a primary electron sink for EEU

RuBisCO catalyzes a reaction between RuBP and CO_2_ that results in the formation of two molecules of 3-phosphoglycerate (3-PGA), with no requirement for reducing equivalents^30^. The reactions that follow, however, require ATP and NAD(P)H. Phosphoglycerate kinase (PGK) catalyzes the phosphorylation of 3-PGA by ATP, which is converted in the reductive phase of the cycle by glyceraldehyde 3-phosphate dehydrogenase (GAPDH) into glyceraldehyde 3-phosphate (G3P). Thus, the CBB cycle, and not RuBisCO directly, is likely the electron sink for EEU. Because *ruBisCO* is the primary autotrophic carboxylase (Fig. 4b) and because form I *ruBisCO* was upregulated during EEU (Fig. 4a), we tested the effect of the lack of *ruBisCO* on this process.

**Fig. 5:**
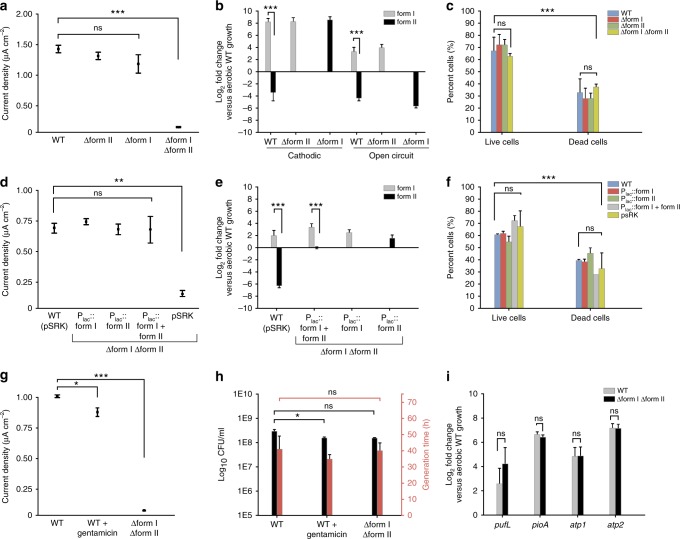
RuBisCO is required for extracellular electron uptake. **a** Endpoint current densities for *ruBisCO* deletion mutants compared to TIE-1 wild-type (WT). Data are means ± s.e.m. of three biological replicates. **b** *ruBisCO* mRNA log_2_ fold change under poised current (cathodic) and no current (open-circuit) conditions for TIE-1 WT and *ruBisCO* deletion mutants. **c** LIVE/DEAD® staining of electrode-attached cells under cathodic conditions. Data are means ± s.e.m. of three biological replicates assayed in triplicate. % represents the percent cells in relation to the total number of cells counted. **d** Endpoint current densities for *ruBisCO* complementation mutants. Data are means ± s.e.m. of three biological replicates. **e** *ruBisCO* mRNA log_2_ fold change under cathodic conditions for TIE-1 WT and *ruBisCO* complementation mutants. **f** LIVE/DEAD® staining of electrode-attached cells under cathodic conditions. Data are means ± s.e.m. of three biological replicates assayed in triplicate. **g** Endpoint current densities under standard conditions (WT) and when treated with gentamicin (WT + gentamicin). Data are means ± s.e.m. of three biological replicates. **h** Log_10_ colony forming units (CFU) and generation time (**h**) of planktonic cells incubated under standard conditions (WT) and when treated with gentamicin (WT + gentamicin). Data are means ± s.e.m. of at least two biological replicates assayed in triplicate. **i** mRNA log_2_ fold change of photosynthetic reaction center (*pufL*), *pio* operon (*pioA*), and ATP synthase homologs (*atp1*, *atp2*) in TIE-1 WT and the *ruBisCO* double mutant. RT-qPCR data are means ± s.e.m. of two biological replicates assayed in triplicate. The *P* values were determined by one-way ANOVA followed by a pairwise test with Bonferroni adjustment (*P < 0.05, **P < 0.01, ***P < 0.0001; ns, not significant). Source data are provided as a Source Data File

We grew WT and the *ruBisCO* double mutant in bulk BESs. We chose this bioelectrochemical format because of the need for more biomass for downstream studies. After ~60 h of incubation in bulk BESs, the peak current density in the WT remained stable at ~ −1.5 µA cm^−2^ (Fig. 5a). The *ruBisCO* double mutant had a 90% reduction in current uptake vs. WT (*P* < 0.0001, one-way ANOVA; Fig. 5a). To assess *ruBisCO* gene expression, we performed reverse transcription quantitative PCR (RT-qPCR) on the planktonic cells. In the WT, form I *ruBisCO* was upregulated ~8-fold with an associated downregulation of form II *ruBisCO* (*P* < 0.0001, one-way ANOVA; Fig. 5b). These expression data in the WT coincide with previous studies on EEU by TIE-1^3^.

The *ruBisCO* mutants did not have a cell viability defect across incubations compared to the WT (*P* = 0.3691, one-way ANOVA; Fig. 5c, Supplementary Figure 6). We also assessed NADH/NAD^+^ and NAD(P)H/NAD(P)^+^ ratios in the *ruBisCO* double mutant and observed that these cells were more reduced under EEU compared to aerobic chemoheterotrophic conditions (Supplementary Figure 7). However, because these cells show very low current uptake (Fig. 5a), these data are difficult to interpret. Additionally, we did not observe a difference in ATP levels in the WT and the *ruBisCO* double mutant planktonic cells during EEU (*P* = 0.2612, one-way ANOVA; Supplementary Figure 8).

Upon complementation of the *ruBisCO* double mutant with form I and/or form II *ruBisCO* (Supplementary Table 4), current uptake reached ~ −0.75 µA cm^−2^, similar to EEU by the WT (Fig. 5d). This was above current uptake levels by the *ruBisCO* double mutant (*P* < 0.01, one-way ANOVA; Fig. 5d). We observed that form I and form II *ruBisCO* were expressed at levels similar to the WT (Fig. 5e). Similar to the *ruBisCO* deletion mutants, the *ruBisCO* complementation mutants did not have a cell viability defect compared to the WT (*P* = 0.0572, one-way ANOVA; Fig. 5f, Supplementary Figure 6).

### RuBisCO deletion does not affect EEU due to a growth defect

To determine whether the EEU defect in the *ruBisCO* double mutant was growth-dependent, we inoculated WT cells into bioreactors containing a sub-lethal concentration of gentamicin to inhibit protein synthesis (Supplementary Figure 9). We observed that gentamicin-treated WT cells accepted 80% more electrons during EEU compared to the *ruBisCO* double mutant (*P* < 0.0001, one-way ANOVA; Fig. 5g). To assess a potential growth defect in the *ruBisCO* double mutant, we harvested the electrodes at the end of the incubations and used 5 mm sections as inoculum for chemoheterotrophic growth. We did not observe a growth defect in the *ruBisCO* double mutant upon re-growth compared to the WT (*P* = 0.8232, one-way ANOVA; Fig. 5h). Planktonic colony forming units (CFUs) for the *ruBisCO* double mutant harvested at the end of incubations in the bulk bioreactors were not different from the WT (*P* = 0.0804, one-way ANOVA; Fig. 5h). These data suggest that the lower EEU activity of the *ruBisCO* double mutant is not due to a growth defect.

We performed gene expression analysis using a set of genes that have been reported to be involved in EEU from electrodes^3^. We first assessed the expression level of the photosynthetic reaction center large subunit (*pufL*). Gene expression analysis showed a ~5-fold upregulation of *pufL* in the *ruBisCO* double mutant, very similar to the WT expression (*P* = 0.0559, one-way ANOVA; Fig. 5i). Because previous mutant studies have shown that the *pioABC* system, a gene operon essential for phototrophic iron oxidation^47^, also has a role in electron uptake^3^, we performed expression analysis of *pioA* in the *ruBisCO* double mutant and the WT. We observed that the expression level of *pioA* in the *ruBisCO* double mutant was not different from the WT (*P* = 0.0759, one-way ANOVA; Fig. 5i).

We also assessed the expression of the systems responsible for energy transduction. The TIE-1 genome contains two F-type ATPases: Atp1 and an “alternate” Atp2. *atp1* showed lower upregulation (~4-fold) than *atp2* (~7-fold) in both the WT and the *ruBisCO* double mutant (Fig. 5i). The WT transcriptomic data corroborate the RT-qPCR data where *atp1* is downregulated during phototrophic growth conditions, including EEU, whereas *atp2* is specifically upregulated during EEU (Supplementary Tables 8, 9). These results suggest that the *atp2* operon plays an important role in ATP synthesis during EEU. Overall, our data suggest that the WT and the *ruBisCO* double mutant do not show any differences in the level of gene expression for critical genes required for EEU, pETC, and energy generation. These data, in conjunction with the lack of ^13^CO_2_ assimilation (Fig. 4b), suggests the *ruBisCO* double mutant cells may be using cellular reserves to stay viable under the conditions tested.

### The CBB cycle is important for phototrophic H_2_ oxidation

**Fig. 6:**
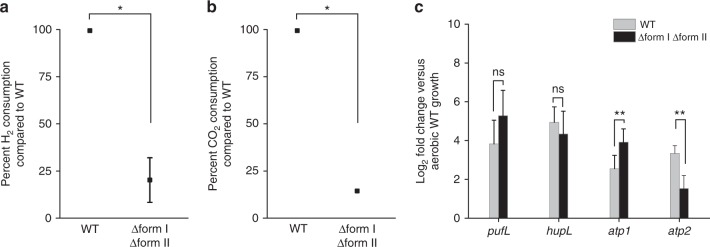
RuBisCO is important for phototrophic hydrogen (H_2_) oxidation. **a** Hydrogen (H_2_) oxidation and **b** carbon dioxide (CO_2_) consumption by the *ruBisCO* double mutant (∆form I ∆form II) as a percent of consumption by TIE-1 wild-type (WT). Data are means ± s.e.m. of two biological replicates assayed in triplicate. **c** mRNA log_2_ fold change of photosynthetic reaction center (*pufL*), NiFe hydrogenase (*hupL*), and ATP synthase homologs (*atp1*, *atp2*) in WT and the *ruBisCO* double mutant. RT-qPCR data are means ± s.e.m. of two biological replicates assayed in triplicate. The *P* values were determined by one-way ANOVA followed by a pairwise test with Bonferroni adjustment (*P < 0.05, **P < 0.01, ***P < 0.0001; ns, not significant). Source data are provided as a Source Data File

The inability of the *ruBisCO* double mutant to take up electrons from solid electrodes suggests that the CBB cycle is the primary electron sink during EEU. This finding underscores that CO_2_ fixation is tightly linked to EEU in these bacteria. In order to probe whether this coupling extends to other growth conditions, we examined the ability of the *ruBisCO* double mutant to oxidize H_2_ under phototrophic conditions. We observed ~80% lower H_2_ consumption in the *ruBisCO* double mutant compared to the WT (*P* < 0.05, one-way ANOVA; Fig. 6a, Supplementary Table 10) with a concomitant reduction in CO_2_ consumption (*P* < 0.05, one-way ANOVA; Fig. 6b, Supplementary Table 10). We also observed an increase in biomass in the WT compared to the *ruBisCO* double mutant during phototrophic H_2_ oxidation (*P* < 0.0001, one-way ANOVA; Supplementary Figures 10, 11). These data suggest that CO_2_ fixation is an important electron sink under photoautotrophic conditions, where electron donors, such as H_2_, are oxidized to provide cellular reducing power.

The *ruBisCO* double mutant might oxidize less H_2_ because gene expression of the uptake hydrogenase^48^ is lower. We therefore assessed the expression of the large subunit of the uptake hydrogenase (*hupL*) in the *ruBisCO* double mutant and found that its expression was not altered compared to WT levels (*P* = 0.3222, one-way ANOVA; Fig. 6c). This suggests that the level of phototrophic H_2_ oxidation between the WT and the *ruBisCO* double mutant should be similar. However, our data show a clear reduction in H_2_ oxidation of ~80% in the mutant strain. We also assessed the expression of *pufL* in the *ruBisCO* double mutant and found no difference in expression vs. the WT (*P* = 0.0753, one-way ANOVA; Fig. 6c). In contrast, *atp1* gene expression was higher in the WT (*P* < 0.01, one-way ANOVA) while *atp2* gene expression was higher in the *ruBisCO* double mutant (*P* < 0.01, one-way ANOVA; Fig. 6c). Our data suggest that the lack of *ruBisCO* affects the ability of TIE-1 to accept electrons from other electron donors such as H_2_.

## Discussion

**Fig. 7:**
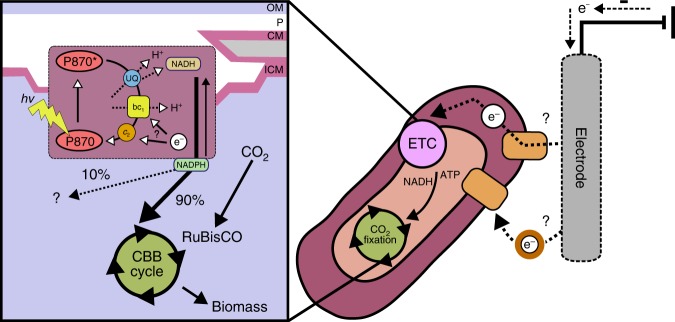
Conceptual model of phototrophic extracellular electron uptake. Extracellular electron uptake is connected to the photosynthetic electron transport chain (pETC) and carbon dioxide (CO_2_) fixation in *R. palustris* TIE-1. The CBB cycle (Calvin-Benson-Bassham) uses RuBisCO and is the primary sink for electrons that enter the photosystem from poised electrodes. The electrons are used by the CBB cycle as NAD(P)H (reduced nicotinamide adenine dinucleotide phosphate) that is exchanged with NADH (reduced nicotinamide adenine dinucleotide) produced via reverse electron flow. For details please read the text. ATP (adenosine triphosphate), e^−^ (electrons), P_870_ (photosystem), P_870_^*^ (excited photosystem), UQ (ubiquinone), *bc*_1_ (cytochrome *bc*_1_), *c*_2_ (cytochrome *c*_2_), H^+^ (protons), *hv* (light), ? (currently unknown), OM (outer membrane), P (periplasm), CM (cytoplasmic membrane) and ICM (inner cytoplasmic membrane)

Microbes have been known to exchange electrons with SPCSs for nearly a century^7^. Although we know the underlying electron transfer pathways and electron sinks employed by microbes that use SPCSs as electron acceptors, these are largely unknown for microbes that use SPCSs as electron donors^4,8^. To fill this knowledge gap, here we used an interdisciplinary approach to study the model EEU-capable microbe *R. palustris* TIE-1. Our data shows that EEU from poised electrodes is connected to pETC and CO_2_ fixation (Fig. 7). We observe that electrons enter the pETC, and eventually these electrons reduce NAD^+^ for CO_2_ fixation via the CBB cycle (Fig. 7). Furthermore, NADH dehydrogenase plays an important role in EEU (Fig. 2) most likely for generation of reducing equivalents for cellular metabolism.

Our inhibitor studies (Fig. 2) and biochemical assays (Fig. 3) suggest that during EEU, electron flow leads to NAD^+^ and NAD(P)^+^ reduction. Because the reduction potential of the electrode in our experiments is lower than that required to reduce NAD^+^/NAD(P)^+^ directly, reverse electron transfer has to occur. The path of reverse electron transfer has been extensively studied in chemolithoautotrophs^34,37,39,49^. In these bacteria, electrons from soluble ferrous iron enter at cytochrome *c*_2_. These electrons can reduce oxygen to generate a PMF for ATP synthesis. The PMF can also be used to drive reverse electron flow from cytochrome *bc*_1_ to NADH dehydrogenase to reduce NAD^+^^34,37,39,49^. NADH dehydrogenase-mediated reverse electron flow has also been observed in *R. capsulatus*^34^. This pathway for electron transfer to NAD^+^ has also been proposed for other anoxygenic phototrophs^50,51^. Our data implies reverse electron flow is also occurring during EEU in TIE-1.

Interestingly, we observe that EEU is reversible in TIE-1 (Fig. 2a, b). Although artificially induced in our system (i.e., only in the presence of antimycin A or CCCP), the reversibility of extracellular electron transfer pathways is broadly observed in bacteria donating electrons to SPCSs^14,27^. For example, *Shewanella oneidensis* MR-1 uses an electron conduit called the Mtr system to transfer electrons to SPSCs^14^. Mtr can also function in reverse to facilitate EEU^14^. The PioAB system (a homolog of the MtrAB system) in TIE-1^47^ plays a role in EEU from poised electrodes^3^. Anoxygenic photoheterotrophs are known to use CO_2_ as an electron sink to maintain redox balance when growing on highly-reduced substrates such as butyrate^52^. In nature, photoheterotrophs may use this reversibility of the EEU pathways and use SPCSs as electron sinks.

SIMS analysis demonstrates CO_2_ fixation is occurring during EEU primarily via RuBisCO (Fig. 4). We observed ^13^C assimilation was identical in surface-attached and planktonic cells within the bulk bioreactors. Furthermore, reactors with planktonic cells have higher current densities versus plankton-free reactors (Supplementary Figure 5) suggesting that they contribute to EEU via an unknown mechanism (Supplementary Figure 12). Previously published work from our laboratory, however, suggests no redox active molecule is detectable in the spent-medium^3^. Our laboratory has also shown that a cathode-driven Fe(II)/Fe(III) redox cycle at +100 mV vs. SHE^10^, is also unlikely.

The *ruBisCO* mutant is impaired in using electron donors such as poised electrodes (Fig. 5) and H_2_ for photosynthesis (Fig. 6). This implies that the cells ability to fix CO_2_ via *ruBisCO* is relayed to the electron transfer machinery that accepts electrons from these electron donors. During EEU we observe both increased *ruBisCO* expression (Fig. 4a) and an increased NAD(P)H/NAD(P)^+^ ratio (Fig. 3b). In *R. palustris* CGA009/10, which is closely related to TIE-1, form I *ruBisCO* is transcriptionally activated in response to elevated NAD(P)H and ATP levels via a regulatory system called CbbRRS^53,54^. These studies suggest that form I RuBisCO may be a sensor of cellular energy and redox balance^53,54^. In TIE-1, the regulatory CbbRRS system may also sense NAD(P)H levels and regulate form I *ruBisCO* expression. Together, this suggests that NAD(P)H is a metabolite that communicates redox status to the CBB cycle by controlling *ruBisCO* expression. This relationship between carbon metabolism and electron transfer may be conserved in other organisms, and thus be broadly relevant in many ecosystems.

Our data highlights that photosynthetic EEU is linked to the CBB cycle for CO_2_ fixation. The link between EEU and the CBB cycle is the reducing equivalents produced via the pETC (Fig. 7). Because the CBB cycle^1^ and EEU^4,5^ are important processes in nature, primary productivity may be attributed to this process. Future studies will focus on quantitative measurements of the prevalence of autotrophic EEU such that EEU-linked CO_2_ fixation can be accounted for in global biogeochemical cycles. EEU from natural SPCSs such as rust might represent a strategy that autotrophic microbes use to access electrons for microbial survival when other electron donors are limiting or otherwise unavailable due to spatiotemporal constraints. Photoautotrophs, which are restricted to the photic zone, are known to exchange electrons with SPCSs, including magnetite^55^. Indeed, studies have shown that SPCSs can potentiate interspecies electron transfer^55–57^. For example, *Geobacter sulfurreducens* can exchange electrons with TIE-1 via mixed valent iron oxides^55^. Furthermore, long distance extracellular electron transfer has been observed by various researchers^7,8^. Although some microbes have evolved specialized membranes to facilitate long distance extracellular electron transfer^58–60^, microbes may also utilize electrically conductive minerals to access electrons in deeper sedimentary zones to overcome spatial separation from electron donors. Because SPCSs are ubiquitous^8,61^, EEU might be used both for microbial growth and survival.

## Methods

### Bacterial strains and culture conditions

All strains used in this study are indicated in Supplementary Table 4. The *Rhodopseudmonas palustris* TIE-1 *ruBisCO* deletion mutants (∆*cbbLS*, *Rpal*_1747-1748; ∆*cbbM*, *Rpal*_5122; and ∆*cbbLS* ∆*cbbM*) were constructed using a suicide plasmid system (Supplementary Table 4)^13^. A complete list of cloning and sequencing primers and restriction enzymes can be found in Supplementary Table 11. *Escherichia coli* strains were routinely cultivated in lysogeny broth (LB; pH 7.0) in 10 mL culture tubes or on LB agar at 37 °C. TIE-1 was pre-grown chemoheterotrophically at 30 °C in YP medium (0.3% yeast extract and 0.3% Bacto peptone) supplemented with 10 mM MOPS pH 7.0 (YPMOPS) in the dark. All growth experiments were carried out at 30 °C unless otherwise noted. All phototrophic growth experiments were carried out with a single 60 W incandescent light bulb at a distance of 25 cm. For anaerobic photoautotrophic growth TIE-1 strains were grown on 80% hydrogen-20% carbon dioxide (H_2_-CO_2_) at ~50 kPa in freshwater medium^62^ (FW) with 20 mM sodium bicarbonate in sterile, sealed, glass serum bottles. For anaerobic photoheterotrophic growth TIE-1 was grown in 10 mL FW medium supplemented with 1 mM acetate or butyrate from stock solutions (100 mM, pH = 7). In all cases where a change in culture medium was required cells were washed three times in basal FW medium post-centrifugation at 5000 × *g*. Bioelectrochemical reactor studies were performed with FW medium lacking exogenous electron donors, and purged with 80–20% nitrogen (N_2_-CO_2_). The complemention experiments were carried out with 1 mM IPTG and 800 µg mL^−1^ gentamicin for plasmid selection. Doubling time was calculated using the equation *g* = ln(2)/*k*, where *k* was determined from the slope of OD_660_ versus time on a log_10_ scale.

### Complementation of *ruBisCO* knockouts

The TIE-1 form I *ruBisCO* (*cbbLS*) and form II *ruBisCO* (*cbbM*) genes were cloned such that the start site overlapped with an NdeI restriction site for cloning into pSRKGm (Supplementary Table 4). A complete list of primers and restriction enzymes used in cloning can be found in Supplementary Table 11. Post-cloning, the *ruBisCO* complementation plasmids were conjugated into the *ruBisCO* double mutant (∆*cbbLS* ∆*cbbM*) using the mating strain *E. coli* S17-1/λpir and selected on 800 µg mL^−1^ YPMOPS agar plates. A single colony was chosen and grown on 1 mM IPTG. Colonies were PCR screened using the primers in Supplementary Table 11. The pSRKGm empty vector was introduced into the WT and the *ruBisCO* double mutant to serve as controls (Supplementary Table 4).

### RNA isolation and RT-qPCR

For bioelectrochemical studies, planktonic cells were sampled in an anaerobic chamber and immedietly mixed 1:1 with RNA*later*® (Qiagen, USA). RNA was extracted using the RNeasy® Mini Kit according to the manufacturer’s recommendations (Qiagen, USA). DNA removal was performed using Turbo DNA-*free*™ Kit (Ambion, USA). RNA samples were tested for purity using PCR. Gene expression analysis of *ruBisCO* was performed using RT-qPCR with the comparative Ct method. Primer efficiencies were determined according to the manufacturers reccommendations. Purified RNA was used to synthesize cDNA with the iScript™ cDNA synthesis kit. *clpX* and *recA* were used as internal standards based on previous studies^3^. Primers for RT-qPCR outlined in Supplementary Table 12 were designed in Primer3 v4.1.0 (http://primer3.ut.ee) using the programs default parameters. The Bio-Rad iTaq™ Universal SYBR® Green Supermix and the Bio-Rad CFX Connect™ Real-Time System Optics ModuleA machine (Bio-Rad Laboratories, Inc., Hercules, CA) were used for all quantitative assays according to the manufacturer’s recommendations.

### Differential expression (RNA-seq) analysis

Transcriptomic data sets were downloaded from NCBI (BioProject: PRJNA417278) and differential expression and statistical analysis was performed. Trimmomatic version 0.36 was used to trim Illumina sequencing reads (threshold = 20) and length filter (min = 60 bp)^63^. Processed reads were mapped to the published *R. palustris* TIE-1 genome using TopHat2 version 2.1.1 and the gff3 annotation file as a guide for sequence alignment^64^. Bowtie 2 version 2.3.3.1 was used to index the reference genome FASTA file^65^. The number of reads mapping to each feature were counted by HTSeq version 0.9.1^66^. Differentially expressed genes were determined in DESEQ2 version 1.16.1 using the HTSeq read counts. To determine if genes were significantly differentially expressed an adjusted *p*-value cutoff of 0.05 was used. Heat maps were drawn in R using ggplot2^67^.

### Quantification of NADH/NAD^+^ and NAD(P)H/NAD(P)^+^ ratios

NADH/NAD^+^ and NAD(P)H/NAD(P)^+^ ratios were quantified using the “high-sensitivity” reagent mixture and sampling procedure^41^. Briefly, two separate 2 mL cell aliquots were sampled in an anaerobic chamber and centrifuged for 1 min at 21,000 × *g* to remove the supernatant. Cell pellets were then resuspended in 200 µL 0.2 M hydrochloric acid (for NAD^+^ and NAD(P)^+^) or sodium hydroxide (for NADH and NAD(P)H) for 10 min at 50 °C, then chilled on ice for 5 min. The reaction was then neutralized dropwise with equal volume 0.1 M acid or base and centrifuged for 5 min at 21,000 × *g*. The supernatant was stored at −80 °C for no more than one week. The enzyme cycling assays were performed on a BioTek Synergy™ HTX 96-well plate reader measuring absorbance at 570 nm^41^. A standard curve of known concentrations of NAD^+^ and NAD(P)^+^ was used to determine the concentration of samples.

### ATP quantitation

ATP was extracted using the boiling water method^68^. Briefly, 2 mL of cells were centrigured at 21,000 × *g* for 1 min and the cell pellet was resuspended in 50 µL boiling sterile-filtered Milli-Q® water and allowed to sit at room temperature for 10 min. Samples were then centrifuged at 21,000 × *g* for 1 min and the supernatant was transferred to fresh microcentrifuge tubes and stored at −80 °C for no more than one week. The ATP Determination Kit (Molecular Probes, Eugene, OR) was used to measure ATP concentrations using a standard curve of known concentrations according to the manufacturers reccomendations. Absorbance was measured at 560 nm. ATP concentrations were normalized to biomass (OD_660_).

### Bulk BES setup and conditions

BESs were configured as previously described^10^. Briefly, FW media (70 mL) was dispensed into sterile, sealed, three-electrode BESs which were bubbled for 60 min with 80%:20% N_2_-CO_2_ to remove oxygen, and pressurized to ~50 kPa. The three electrodes were configured as follows: graphite working electrodes were ~3.2 cm^2^; reference eletrodes (Ag/AgCl) were submerged in 3 M KCl; and counter electrodes were composed of 5 cm^2^ platinum foil. Working electrodes were poised at +100 mV versus SHE using a multichannel potentionstat (Gamry Instruments, Warmister, PA) and operated continuously with a single 60 W incandescent light bulb at 26 °C. Data were collected every 1 min using the Gamry Echem Analyst™ (Gamry Instruments, Warmister, PA) software package. The biomass (OD_660_) of inoculated BESs was monitored with a BugLab Handheld OD Scanner (Applikon Biotechnology, Inc., Foster City, CA).

### Quantification of live/dead bacteria on electrodes

Graphite electrodes were washed three times with anoxic 1× phosphate-buffered saline (PBS) to remove unattached cells in an anaerobic chamber. Sections of the electrode were cut with a sterile razor blade and immediately placed in sterile microfuge tubes containing anoxic 1× PBS. Prior to imaging, the electrode was immersed in LIVE/DEAD® stain (10 µM SYTO 9 and 60 µM propidium iodide, L7012, Life Technologies) and incubated for 15 min in the dark. Samples were then placed in 1× PBS in a glass bottom Petri dish (MatTek Corporation, Ashland, MA). For imaging biofilms in the intact µ-BEC, LIVE/DEAD® stain was flowed into the µ-BEC and allowed to incubate for 15 min in the dark. The excess stain was washed with sterile anoxic 1× PBS. Electrodes were imaged on a Nikon A1 inverted confocal microscope using 555 and 488 nm lasers and a ×100 objective (Washington University in St. Louis Biology Department Imaging Facility). Attached cells were quantified in Fiji v1.0 (https://fiji.sc) using the analysis pipeline described below. Briefly, images (*n* = 3) were inverted then converted to a 1-bit image by auto-thresholding. The “Watershed” tool was then applied to separate object edges. The “Analyze Particles” tool was used to generate cell counts for each image based on an area range (min = 16 pixels, max = 210 pixels) that was empirically determined from manually masking 100 cells. The red and green channels were split, and the “Analyze Particles” tool was used to count bacteria on each image (1024 × 1024 pixels).

### Micro-bioelectrochemical cell (µ-BEC) setup and conditions

The µ-BECs were assembled from polymer fluidic layers, indium tin oxide (ITO) coverslips, and a glass layer with integrated reference and counter electrodes. Inlet, outlet, and connecting channels were laser cut into a 40 mm × 12.25 mm × 254-µm thick acetal polyoxymethylene (POM) adhesive tape. Four 4 mm diameter reaction chambers were cut into a second 127-µm thick acetal POM tape, aligned, and bonded to the channel layer using a pressure-sensitive acrylic adhesive. Prior to assembly, 1-mm diameter inlet/outlet holes were drilled into Borofloat® 33 1.75-mm thick glass capping layer (Schott AG, Mianz, Germany). Five hundred-micrometers of deep grooves were diced into the glass above the chamber midlines to locate 250-µm silver and platinum wires used for reference (RE) and counter (CE) electrodes, respectively (Xi'an Yima Opto-electrical Technology Co., Ltd, Shaanxi, China). Each 1.6 μL (0.125 cm^2^) well was enclosed by a 6 mm × 10 mm × 170-µm thick ITO-coated coverslip (30–60 Ω) (SPI supplies, West Chester, PA) to serve as the working electrode. Inlet and outlet tubes (Saint-Gobain TYGON® b-44-3; 1/16" ID × 1/8" OD) (United States Plastic Corp., Lima, OH) were attached on the glass capping layer and the 1/16" tube ends were capped with male/female luer lock fittings (World Precision Instruments, Sarasota, FL). Microbial samples were injected into the µ-BEC using a FLOW EZ™ Fluigent Microflow Controller (Le Kremlin-Bicêtre, France) with 5 kPa 80–20% N_2_-CO_2_. Microbial cells were incubated in µ-BECs with working electrodes poised at +100 mV vs. SHE for ~120 h under illuminated conditions with a single 60 W incandescent light bulb at a distance of 25 cm to establish biofilms. Once we obtained stable current densities under illuminated conditions (~ −100 nA cm^−2^), planktonic cells were washed out of the system with microfluidic control and biofilms were immedietly treated with chemical inhibitors under dark conditions. Light “on-off” experiments were subsequently carried out at an interval of 10 s for a total of 200 s. Microfluidic flow was not applied during electrochemical data collection.

### Analytical techniques

In order to quantify the amounts of H_2_ and CO_2_ consumption during photoautotrophic growth with H_2_, gas concentrations in headspace at the initial and final time points were measured. Twenty microliters of gas sample from the headspace was withdrawn using a Hamilton^TM^ gas-tight syringe and analyzed using a Tracera GC-BID 2010 Plus, (Shimadzu Corp., Japan) equipped with an Rt^®^-Silica BOND PLOT Column (30 m × 0.32 mm; Restek, USA). Based on the measured partial pressures of H_2_ and CO_2_, their concentrations in headspace (moles of gas) were calculated using the ideal gas law (PV = *n*RT).

### Secondary ion mass spectrometry (SIMS)

For planktonic assessments, 2 mL of cells were harvested from the bulk BESs and centrifuged at 4000 × *g* for 10 min. For biofilm assessments, cells were manually dislodged from the electrode by scraping with a sterile razor and resuspended in 950 µL of 1× PBS. Cells were then fixed with 50 µL of 20% paraformaldehyde fixative to a 1% final concentration and incubated at 4 °C for 24 h. After incubation, cells were pelleted by centrifugation, and washed with 1× PBS buffer twice to remove any residual fixative. Lastly, the cells were resuspended in 500 µL 100% ethanol and stored at −20 °C. Carbon isotopic compositions of individual cells were measured on a Cameca IMS 7f-GEO (Ametek Inc., USA) secondary ion mass spectrometer. Areas of interest (~100 µm^2^) were selected via scanning ion imaging, using the criterion of maximizing cell density, without compromising unambiguous individual cell identification. Scanning ion images of ^12^C^14^N^−^ were used for this step, which was preceded by several minutes of pre-sputtering, in order to overcome the surface ion-yield transient region and achieve steady state secondary ion yield. Note that for biological specimens, nitrogen is monitored as CN^−^ which provides a strong, unambiguous signal with which to locate the microbes^69^. Nominal primary ion settings were a 1.5-µm diameter, 20-keV net impact energy, 10 pA Cs^+^ beam rastered over a square area 100 microns per side. ^12^C^−^ and ^13^C^−^ scanning ion images were acquired, sequentially, using magnet switching and a single electron multiplier (EM) detector. In order to avoid EM saturation or aging, the instantaneous secondary ion count rate was restricted to <3 × 10^5^ counts per second. A magnetic field settling time of 1 s was included prior to each new image acquisition. The acquisition time per image was nominally 5 s for ^12^C^−^ and 55 s for ^13^C^−^. Image acquisition cycling continued until most cellular material was sputtered through (typically between 2 and 4 h). Between two and six fields of view were measured for each sample, depending on cell spacing. For these specimens, the most egregious isobaric interference was ^12^C^1^H^−^, which required a mass resolving power (MRP) of 2909 (M/dm) to achieve mass peak separation from ^13^C^−^. Therefore, the entrance and exit slits were set to achieve a flat-topped peak with MRP = 3000.

### SIMS data analysis

Each region of interest (ROI; i.e., one individual cell) was selected using Cameca WinImage software (Ametek Inc., USA), and all count rates were exported for all ROIs for all cycles. EM dead time and quasi-simultaneous arrival corrections (QSA) were applied^70^. Note that these corrections made a relative change to the corrected ratio from the raw data ratio of only ~0.5% and ~0.2%, respectively. For each field of view, isotope ratios for all ROIs were plotted against cycle number. Based on numerous ‘blank’ analyses of unlabeled microbes, isotope ratios are not statistically ‘normally’ distributed around the mean value as the cell is sputtered through, being skewed at the onset of sputtering (despite pre-loading with Cs) and also when the cell is almost consumed. However, excluding these cycles, histograms of percent deviation from natural abundance of populations of ‘blank’ cells are, indeed, statistically normal, with a typical *relative* standard deviation < 1% (1 s, for >100 cells). (Note that a 1% *relative* standard deviation indicates, for example, that a 20% measured label isotope ratio increase would have a standard deviation of 0.2%). Once the cycles for each field of view were chosen, the ratios were averaged across those cycles for each region of interest. The data were then translated to deviations from unlabeled. For each test, a reference ratio, that is, the mean R^13^C (i.e., ^13^C/^12^C) of the unlabeled data set, is calculated. Then all ratios in that test were recalculated as ‰ (permil, or part per thousand) deviations from the unlabeled mean using the equation δ^13^C_test_ = (R^13^C_test_/R^13^C_ref_−1)*1000 with Microsoft® Excel.

### Electron transport chain inhibitors

Stock solutions (100×) of rotenone, antimycin A, and carbonyl cyanide *m*-chlorophenyl hydrazine (CCCP) (MilliporeSigma, USA) were solubilized in 100% DMSO and stored as aliquots at −20 °C for no more than one day before use. For µ-BEC experiments, the stock solutions were suspended in 1× PBS before use.

### Statistical analysis

All statistical analyses (Student’s *t*-test, one-way ANOVA with Bonferroni adjustment) were performed with Microsoft® Excel “Data Analysis” tools.

### Reporting summary

Further information on experimental design is available in the Nature Research Reporting Summary linked to this article.

## Supplementary information

Supplementary Information

Reporting Summary

Source Data

## Data Availability

All data in this study are available from the corresponding authors upon request. The source data underlying Figs. 1d, 2a–b, 3a–d, 4a–c, 5a–i, 6a–c; Supplementary Figs. 2a–d, 3, 4, 5d–e, 6a-i, 7a–b, 8–11; and Supplementary Tables 1–3 and 5–7 and 10 are provided as a Source Data file. Sequencing reads used for differential expression analysis are available under BioProject PRJNA417278.
