# Supplementary material for: Phototrophic extracellular electron uptake is linked to carbon dioxide fixation in the bacterium Rhodopseudomonas palustris
Source: Nat Commun. 2019 Mar 22;10:1355. doi: 10.1038/s41467-019-09377-6 (PMC6430793; doi:10.1038/s41467-019-09377-6)
Supplement: Supplementary file 1 — Supplementary Information [file 41467_2019_9377_MOESM1_ESM.pdf]

Supplementary Information

Phototrophic extracellular electron uptake is linked to carbon dioxide fixation in the bacterium *Rhodopseudomonas palustris*

Guzman *et al.*

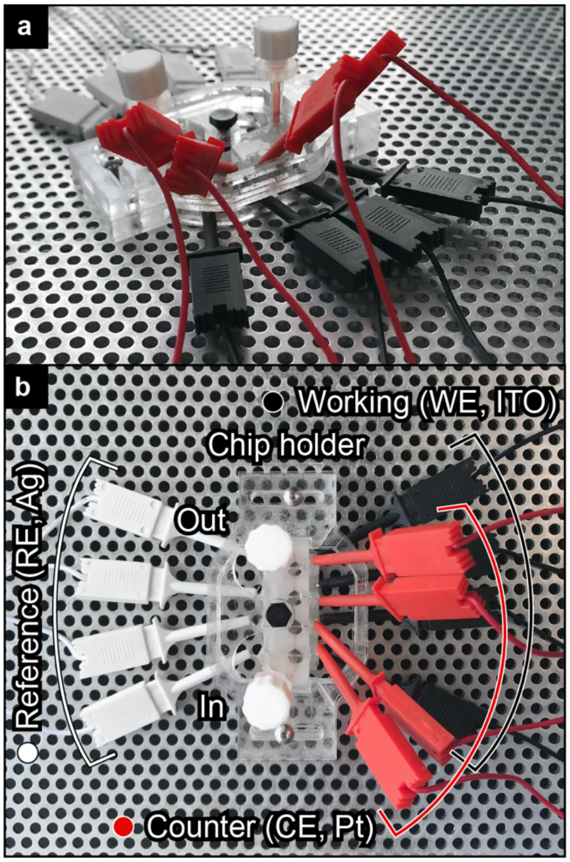

Supplementary Figure 1. Micro-bioelectrochemical cell (μ-BEC). (**a**) Instrument assembly with inlet/outlet capped for incubation. (**b**) Top-down view of four-chamber μ-BEC configuration shown with reference electrode (RE), counter counter (CE), and working electrode (WE) leads connected to integrated silver (Ag) and platinum (Pt) wires, and indium tin oxide (ITO) coverslips (WE).

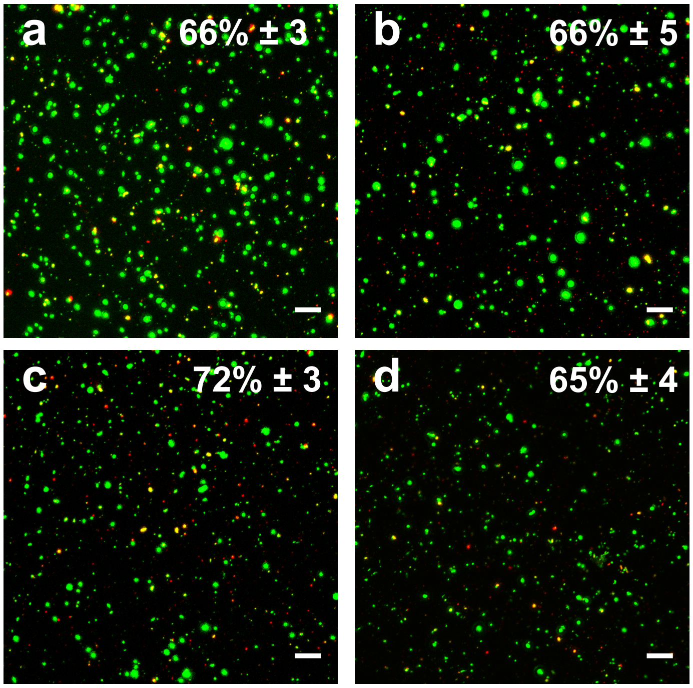

Supplementary Figure 2. Fluorescent micrographs of *Rhodopseudomonas palustris* TIE-1 wild-type (WT) biofilms in μ-BEC. Representative confocal micrographs of TIE-1 biofilms attached to the cathode and stained with LIVE/DEAD® dyes. (**a**) Untreated control, (**b**) antimycin A, (**c**) carbonyl cyanide *m*-chlorophenyl hydrazone (CCCP), and (**d**) rotenone treated cells. Data are the mean percentages of live cells ± s.e.m. of three biological replicates assayed in triplicate. All cells in the field of view were counted. Image manipulation and cell counts were performed in Fiji v1.0 (see Methods). Scale bars are 10 μm. Source data are provided as a Source Data File.

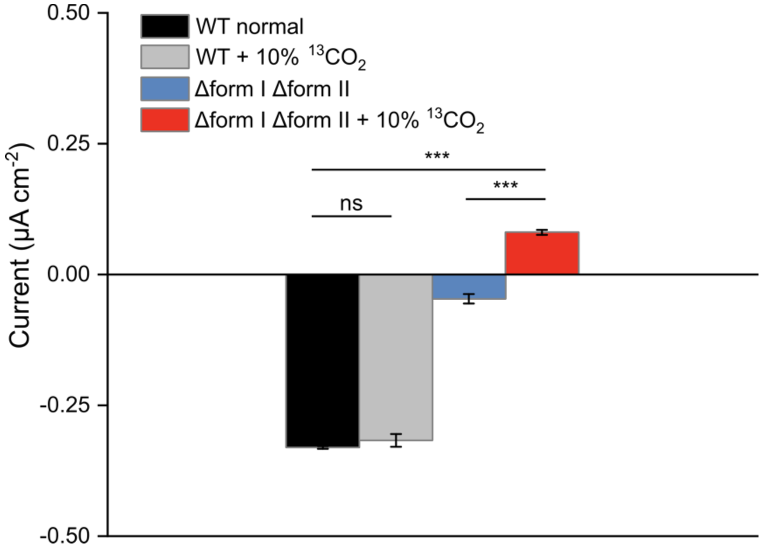

Supplementary Figure 3. Average current densities for *R. palustris* TIE-1 cells harvsted for secondary ion mass spectrometry studies. Current uptake for *R. palustris* TIE-1 WT and the *ruBisCO* double mutant (∆form I ∆form II) after 60 h incubations in bulk bioelectrochemical cells (BECs) with and without 10% $^{13}CO_2$. Data are means ± s.e.m. of current passed over 60 h with a 10 second interval between measurements (number of measurements per condition, n = 25527). The P values were determined by one-way ANOVA followed by a pairwise test with Bonferroni adjustment (*P < 0.05, **P < 0.01, ***P < 0.0001; ns, not significant). Source data are provided as a Source Data File.

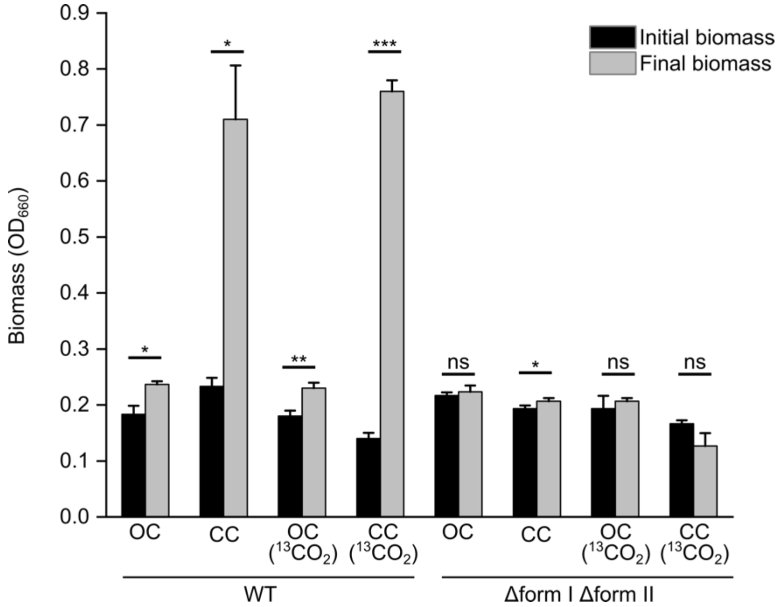

Supplementary Figure 4. Planktonic cell growth of *R. palustris* TIE-1 wildtype (WT) and the *ruBisCO* double mutant (∆form I ∆form II) harvested for secondary ion mass spectrometry. Initial and final optical density (OD_{660}) in bulk BECs after 60 h incubations under open-circuit (OC, reactor not passing current) and standard closed-circuit (CC, reactors passing current) conditions with and without 10% $^{13}CO_2$. Data are means ± s.e.m. of three technical replicates. The *P* values were determined by one-way ANOVA followed by a pairwise test with Bonferroni adjustment (**P* < 0.05, ***P* < 0.01, ****P* < 0.0001; ns, not significant). Source data are provided as a Source Data File.

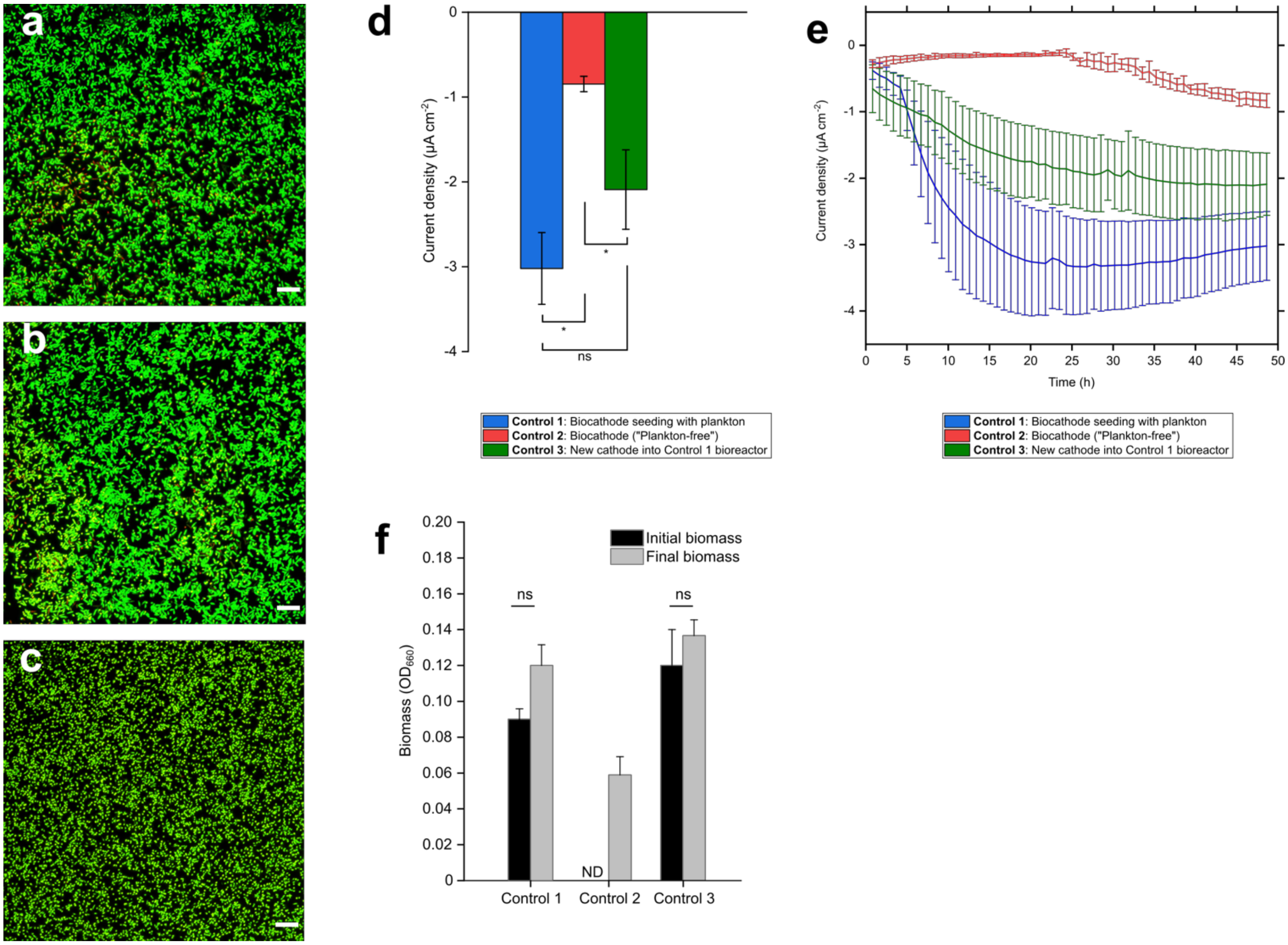

Supplementary Figure 5. *R. palustris* TIE-1 wild-type (WT) biocathode seeding in plankton-free bioreactors. Representative confocal micrographs of TIE-1 biofilms attached to cathode and stained with LIVE/DEAD® dyes. (**a**) "Control 1" biocathode. (**b**) "Control 2" biocathode. (**c**) "Control 3" biocathode. Scale bars are 10 μM. "Control 1" bioreactors were seeded with planktonic cells to generate the biocathodes that were then installed into the "Control 2" bioreactors. "Control 2" bioreactors are plankton-free and contain only biocathodes (with fresh media). "Control 3" bioreactors are the initial "Control 1" bioreactors replaced with new, cell-free cathodes (media was not replaced). (**d**) Mean current densities after 48-hour incubations. (**e**) Mean current density over time. (**f**) Initial and final optical density (OD_{660}) of bioreactors. Data are means ± s.e.m. of three biological replicates. The *P* values were determined by one-way ANOVA followed by a pairwise test with Bonferroni adjustment (**P* < 0.05, ***P* < 0.01, ****P* < 0.0001; ns, not significant). ND (not detectable). Source data are provided as a Source Data File.

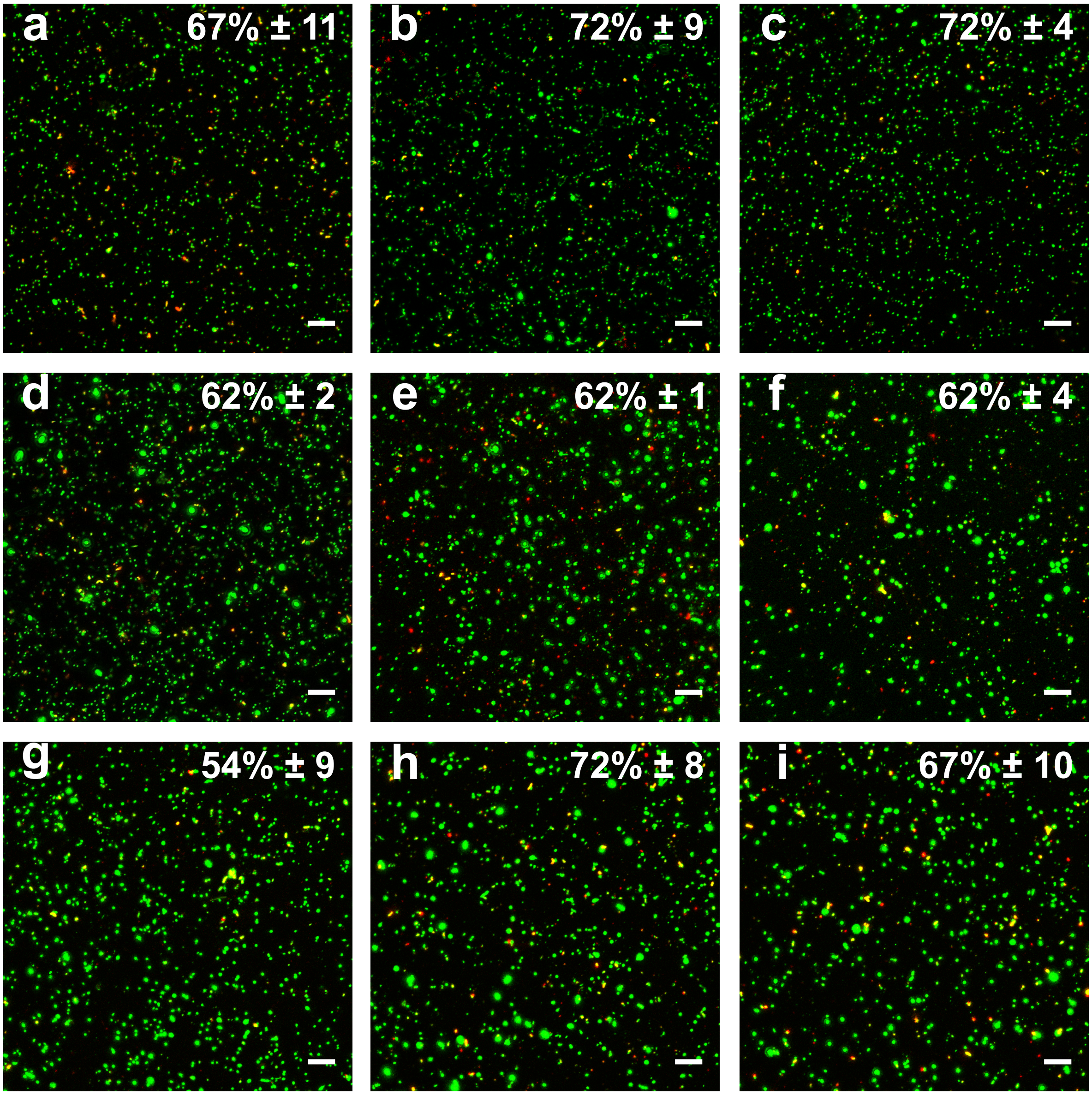

Supplementary Figure 6. Confocal micrographs of *R. palustris* TIE-1 wild-type (WT) and *ruBisCO* deletion and complementation mutant biofilms. Representative images of biofilms attached to cathode in bulk BECs and stained with LIVE/DEAD® dyes: (**a**) WT, (**b**) Δform I, (**c**) Δform II, (**d**) Δform I Δform II, (**e**) WT (pSRK), (**f**) Δform I Δform II (P_{lac}::form I), (**g**) Δform I Δform II (P_{lac}::form II), (**h**) Δform I Δform II (P_{lac}::form I + form II), (**i**) Δform I Δform II (pSRK). Data are the mean percentages of live cells ± s.e.m. of three biological replicates assayed in triplicate. All cells in the field of view were counted. Image manipulation and cell counts were performed in Fiji v1.0 (see Methods). Scale bars are 10 μm. Source data are provided as a Source Data File.

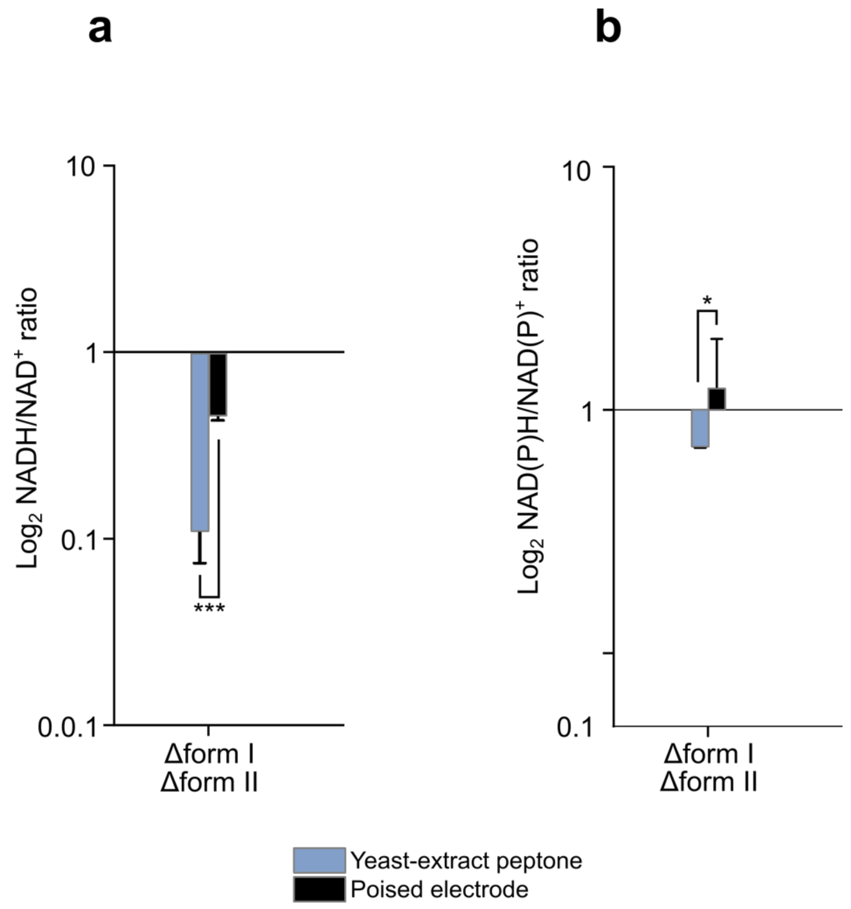

Supplementary Figure 7. $NADH/NAD^+$ and $NAD(P)H/NAD(P)^+$ ratios from *R. palustris* TIE-1 *ruBisCO* double mutant (∆form I ∆form II). (**a**) Log_2 $NADH/NAD^+$ and (**b**) $NAD(P)H/NAD(P)^+$ ratios for *ruBisCO* double mutant cells. Data are means ± s.e.m. of three biological replicates assayed in triplicate. The *P* values were determined by one-way ANOVA followed by a pairwise test with Bonferroni adjustment (*$P < 0.05$, **$P < 0.01$, *** $P < 0.0001$; ns, not significant). Source data are provided as a Source Data File.

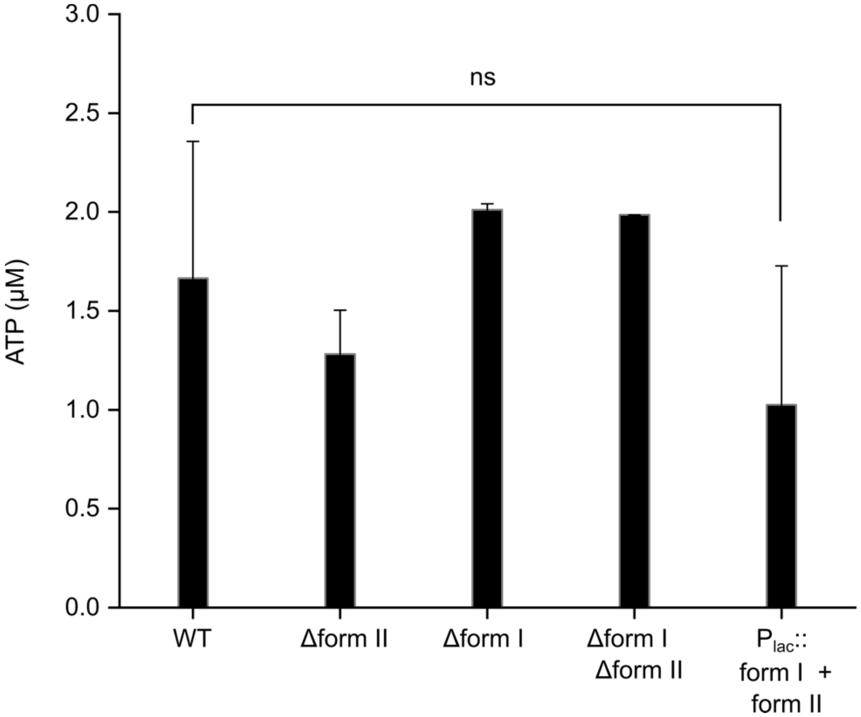

Supplementary Figure 8. ATP quantitation from planktonic *R. palustris* TIE-1 wild-type (WT) and *ruBisCO* deletion and complementation. ATP levels from TIE-1 cells after 60 h incubations in bulk BECs. Data are means ± s.e.m. of at least two biological replicates assayed in triplicate. The *P* values were determined by one-way ANOVA followed by a pairwise test with Bonferroni adjustment (*$P < 0.05$, **$P < 0.01$, ***$P < 0.0001$; ns, not significant). Source data are provided as a Source Data File.

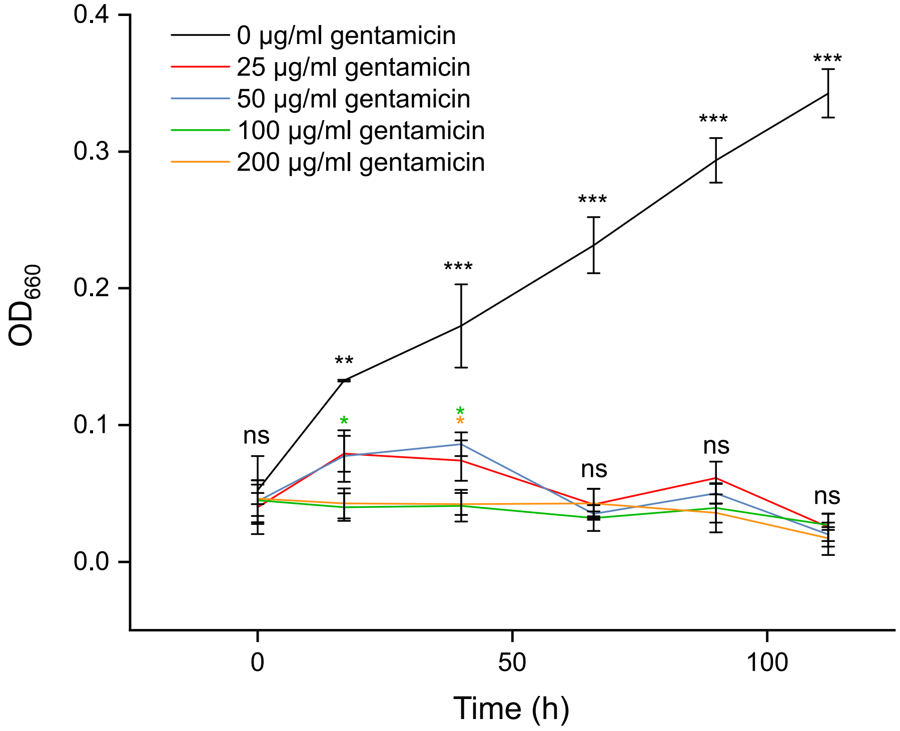

Supplementary Figure 9. Minimum inhibitory concentration (MIC) determination for photoautotrophically-grown *R. palustris* TIE-1 wild-type (WT). Optical density (OD_{660}) during photoautotrophic growth on 80% hydrogen:20% carbon dioxide (H_2:CO_2) with increasing concentrations of gentamicin. Data are means ± s.d. of at least two biological replicates assayed in triplicate. The *P* values were determined by one-way ANOVA followed by a pairwise test with Bonferroni adjustment (*$P < 0.05$, **$P < 0.01$, ***$P < 0.0001$; ns, not significant). Source data are provided as a Source Data File.

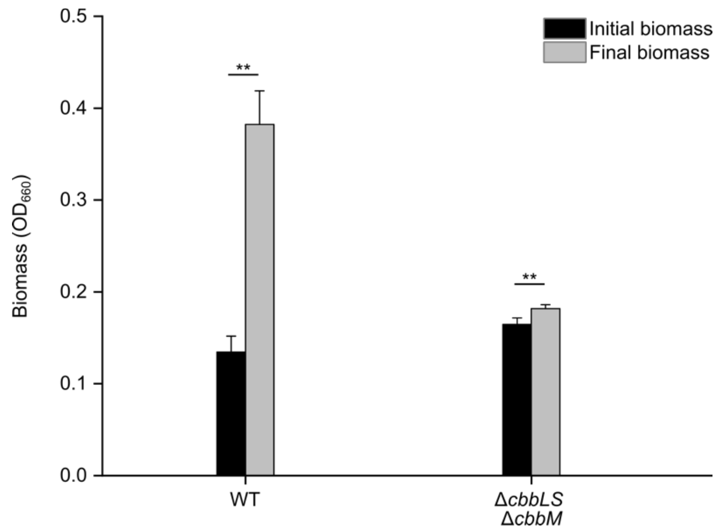

Supplementary Figure 10. Planktonic cell growth of *R. palustris* TIE-1 wild-type (WT) and *ruBisCO* double mutant (∆form I ∆form II) during photoautotrophic growth on H_2:CO_2. Initial and final optical density (OD_{660}) from cells used for quantification of H_2 and CO_2 consumption. Data are means ± s.e.m. of three biological replicates assayed in triplicate. The *P* values were determined by one-way ANOVA followed by a pairwise test with Bonferroni adjustment (*$P < 0.05$, **$P < 0.01$, ***$P < 0.0001$; ns, not significant). Source data are provided as a Source Data File.

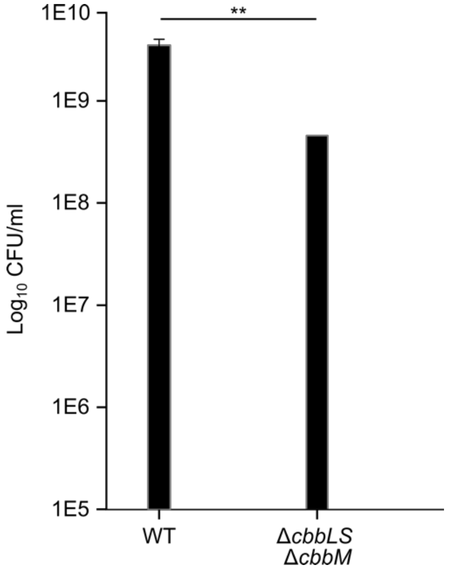

Supplementary Figure 11. Log_{10} colony forming units (CFU) per mL during photoautotrophic growth with H_2. Log_{10} colony forming units of TIE-1 wild-type (WT) and *ruBisCO* double mutant planktonic cells. Cells were plated aerobically onto rich media at the end of incubations that typically lasted 60 hours. Data are means ± s.e.m. of three biological replicates. The *P* values were determined by one-way ANOVA followed by a pairwise test with Bonferroni adjustment (**P* < 0.05, ***P* < 0.01, ****P* < 0.0001; ns, not significant). Source data are provided as a Source Data File.

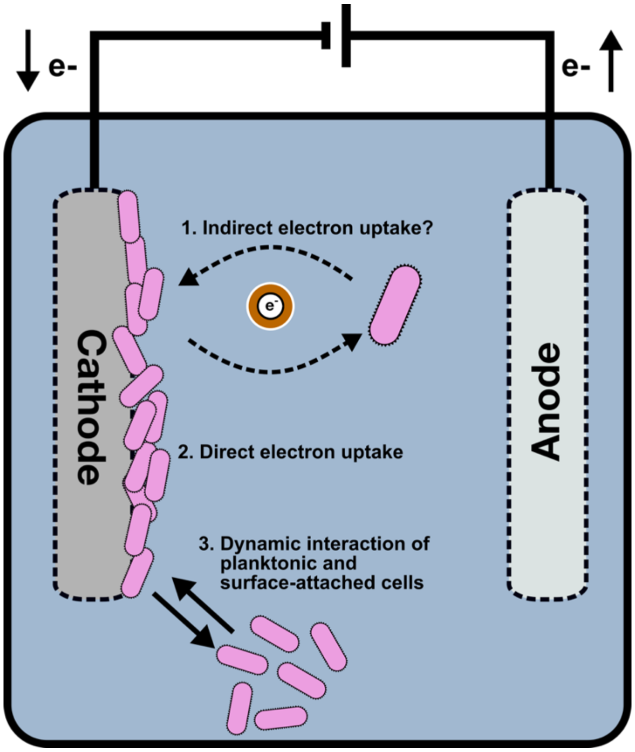

Supplementary Figure 12. Potential mechanisms involved in extracellular electron uptake (EEU) from a poised electrode by planktonic cells. (1) Indirect electron uptake via a soluble redox active compound; (2) Direct electron uptake; (3) Dynamic interaction of planktonic and surface-attached cells.

Supplementary Table 1. Average maximum current density under light and dark conditions with antimycin A treatment in μ-BECs for *R. palustris* TIE-1 wild-type (WT). Data are means ± s.e.m. of three biological replicates. The *P* values were determined by pairwise *t*-test. *P*-values across rows are for comparisons between light and dark treatments. *P*-values in columns are for comparisons between untreated and treated reactors. Source data are provided as a Source Data File.

	**Maximum current density (nA cm^{-2})**			**Coulombs (C)**		
	Light	Dark	*P* (Light vs. Dark)	Light	Dark	*P* (Light vs. Dark)
Untreated control	-85.5 ± 21.7	-9.70 ± 3.59	*P*<0.0001	-121 ± 6.12	-23.5 ± 1.14	*P*<0.0001
100 μM antimycin A	12.5 ± 1.34	-3.46 ± 1.80	*P*<0.0001	4.74 ± 3.34	-2.02 ± 1.14	*P*<0.0001
P (untreated vs. 100 μM antimycin A)	*P*<0.0001	*P*=0.0006		*P*<0.0001	*P*<0.0001	

Supplementary Table 2. Average maximum current density under light and dark conditions with carbonyl cyanide *m*-chlorophenyl hydrazine (CCCP) treatment in µ-BECs for *R. palustris* TIE-1 wild-type (WT). Data are means ± s.e.m. of three biological replicates. The *P* values were determined by pairwise *t*-test. *P*-values across rows are for comparisons between light and dark treatments. *P*-values in columns are for comparisons between untreated and treated reactors. Source data are provided as a Source Data File.

	**Maximum current density (nA cm^{-2})**			**Coulombs (C)**		
	Light	Dark	*P* (Light vs. Dark)	Light	Dark	*P* (Light vs. Dark)
Untreated control	-114 ± 21.7	-17.5 ± 3.41	*P*<0.0001	-119 ± 3.22	-9.65 ± 1.03	*P*<0.0001
25 µM CCCP	21.2 ± 9.13	-18.4 ± 14.01	*P*<0.0001	6.73 ± 4.31	-7.84 ± 2.44	*P*<0.0001
100 µM CCCP	19.3 ± 5.46	-12.9 ± 6.40	*P*<0.0001	18.3 ± 3.74	0.740 ± 5.21	*P*<0.0001
P (untreated vs. 25 µM CCCP)	*P*<0.0001	*P*=0.8666		*P*<0.0001	*P*=0.0025	
P (untreated vs. 100 µM CCCP)	*P*<0.0001	*P*=0.0906		*P*<0.0001	*P*<0.0001	

Supplementary Table 3. Average maximum current density under light and dark conditions with rotenone treatment in μ-BECs for *R. palustris* TIE-1 wild-type (WT). Data are means ± s.e.m. of three biological replicates. The *P* values were determined by pairwise *t*-test. *P*-values across rows are for comparisons between light and dark treatments. *P*-values in columns are for comparisons between untreated and treated reactors. Source data are provided as a Source Data File.

	**Maximum current density (nA cm^{-2})**			**Coulombs (C)**		
	Light	Dark	*P* (Light vs. Dark)	Light	Dark	*P* (Light vs. Dark)
Untreated control	-94.7 ± 3.61	-12.0 ± 4.02	*P*<0.0001	-120 ± 3.73	-16.7 ± 3.73	*P*<0.0001
25 μM rotenone	-71.8 ± 2.02	-25.2 ± 1.60	*P*<0.0001	-73.5 ± 2.32	-38.9 ± 3.07	*P*<0.0001
50 μM rotenone	-60.3 ± 1.81	-19.5 ± 4.94	*P*<0.0001	-64.6 ± 5.67	-34.3 ± 3.42	*P*<0.0001
100 μM rotenone	-41.6 ± 4.55	-25.2 ± 0.820	*P*<0.0001	-60.7 ± 1.88	-26.7 ± 2.41	*P*<0.0001
P (untreated vs. 25 μM rotenone)	*P*<0.0001	*P*<0.0001		*P*<0.0001	*P*<0.0001	
P (untreated vs. 50 μM rotenone)	*P*<0.0001	*P*=0.0050		*P*<0.0001	*P*<0.0001	
P (untreated vs. 100 μM rotenone)	*P*<0.0001	*P*<0.0001		*P*<0.0001	*P*<0.0001	

Supplementary Table 4. Strains and plasmids used in this study.

Strain or plasmid	Genotype and use	Source
***E. coli* strains**		
WM6026	[*lacI^q rrnB3* Δ*lacZ*4787 *hsdR514*Δ*araBAD567* Δ*rhaBA D568 rph-1 att*I::pAE12(Δ*ori*R6K-*cat*::Frt5) Δ*endA*::Frt *uidA*(ΔMluI)::*pir att*HK::pJK1006Δ(*ori*R6K-*cat*::Frt5;*trfA*::Frt)]. Donor strain for conjugation.	W. Metcalf, University of Illinois, Urbana-Champaign
DH10B	F$^-$ *endA1 recA1 galE15 galK16 nupG rpsL ΔlacX74* Φ80*lacZΔM15 araD139 Δ(ara,leu)7697 mcrA Δ(mrr-hsdRMS-mcrBC)* λ^-. Used as standard cloning strain.	1
***R. palustris* strains**		
TIE-1	Wild-type (WT). Isolated from Woods Hole, MA.	2
AB135	Δ*cbbLS* Δ*cbbM* (Rpal_1747- Rpal_1748, Rpal_5122)	This study
AB136	Δ*cbbLS* (Rpal_1747)	This study
AB143	Δ*cbbM* (Rpal_5122)	This study
AB114	WT TIE-1 with pSRKGm	This study
AB142	AB135 with pSRKGm	This study
AB138	AB135 complemented with pAB721	This study
AB139	AB135 complemented with pAB709	This study
AB140	AB135 complemented with pAB720	This study
**Plasmids**		
pAB709	pSRKGm with Rpal_1747-1748 (*cbbLS*) cloned into *Nde*I and *Spe*I sites using primers cbbL-For/cbbLS-Rev.	This study
pAB720	pSRKGm with Rpal_5122 (*cbbM*) cloned into *Spe*I and *Sma*I sites using primers pSRKGm-CbbM-For4-AclI-NdeI/pSRKGm-CbbM-Rev3-AclI-SmaI.	This study
pAB721	pSRKGm with Rpal_1747-1748 (*cbbLS*) and Rpal_5122 (*cbbM*). pAB720 insert was digested and cloned into the *Spe*I and *Sma*I sites of pAB709.	This study
pSRKGm	Complementation plasmid modified from pBBR1MCS-5; Gm^R.	3
pJQ200KS	*sacB*, Gm^R. Suicide vector.	4
pAB621	1 kilobase (kb) upstream and 1 kb downstream of the WT *Rhodospeudmonas* palustris TIE-1 *cbbLS* gene cloned into the *Not*I and *Bam*HI sites of pJQ200KS using primers Rpal_1747_upfor/Rpal_1747_uprev (upstream of *cbbL*) and Rpal_1748_dnfor/ Rpal_1747_dnrev (downstream of *cbbS*).	This study
pAB622	1 kilobase (kb) upstream and 1 kb downstream of the WT *Rhodospeudmonas* palustris TIE-1 *cbbM* gene cloned into the *Not*I and *Bam*HI sites of pJQ200KS using primers Rpal_5122upforNotI/Rpal_5122uprev (upstream of *cbbM*) and Rpal_5122dnfor/ Rpal_5122dnrev (downstream of *cbbM*).	This study

Supplementary Table 5. Doubling time (hours) for aerobic chemophotoheterotrophic, photoheterotrophic (with butyrate and acetate), and photoautotrophic growth by *R. palustris* TIE-1 wild-type (WT) and *ruBisCO* deletion mutants. Data are means ± s.d. of three biological replicates. Source data are provided as a Source Data File.

Growth condition	WT	Δform I	Δform II	Δform I Δform II
Yeast-extract peptone	3.1 ± 0.11	3.6 ± 0.17	4.6 ± 0.95	3.2 ± 0.26
Acetate	5.7 ± 1.1	13 ± 1.4	8.8 ± 1.5	23 ± 0.7
Butyrate	4.6 ± 0.17	4.9 ± 0.29	4.9 ± 0.84	30 ± 2.5
Phototrophic H_2 oxidation	4.3 ± 0.02	4.3 ± 0.01	8.7 ± 0.05	No growth

Supplementary Table 6. Average delta $^{13}C/^{12}C$ ratio values for *R. palustris* TIE-1 wild-type (WT). Data are means ± s.e.m. of at least 25 cells. “Non-poised” refers to open-circuit conditions. “Plankton” are free-living cells sampled from the bioreactors, whereas “electrode” refers to biofilms attached to the cathode (i.e. surface-attached cells). Source data are provided as a Source Data File.

	**Condition**	**Current**	**$^{13}CO_2$**	**Delta $^{13}C/^{12}C$**
1	Control 1 (electrode non-poised)	No	No	0.20 ± 0.19
2	Control 2 (electrode poised)	Yes	No	-0.29 ± 0.21
3	Control 3 (electrode non-poised)	No	Yes	14.4 ± 2.67
4	Test condition 1 (electrode poised)	Yes	Yes	150 ± 2.81
5	Control 4 (plankton non-poised)	No	No	1.55 ± 1.21
6	Control 5 (plankton poised)	Yes	No	3.01 ± 1.95
7	Control 6 (plankton non-poised)	No	Yes	46.4 ± 3.22
8	Test condition 2 (plankton poised)	Yes	Yes	151 ± 2.72

Supplementary Table 7. Average delta $^{13}C/^{12}C$ ratio values for *R. palustris* TIE-1 *ruBisCO* double mutant (∆form I ∆form II). Data are means ± s.e.m. of at least 25 cells. “Non-poised” refers to open-circuit conditions. “Plankton” are free-living cells sampled from the bioreactors, whereas “electrode” refers to biofilms attached to the cathode (i.e. surface-attached cells). Source data are provided as a Source Data File.

	**Condition**	**Current**	**$^{13}CO_2$**	**Delta $^{13}C/^{12}C$**
1	Control 1 (electrode non-poised)	No	No	ND
2	Control 2 (electrode poised)	Yes	No	0.00 ± 0.630
3	Control 3 (electrode non-poised)	No	Yes	ND
4	Test condition 1 (electrode poised)	Yes	Yes	5.91 ± 2.80
5	Control 4 (plankton non-poised)	No	No	-1.57 ± 0.220
6	Control 5 (plankton poised)	Yes	No	-1.40 ± 0.240
7	Control 6 (plankton non-poised)	No	Yes	18.6 ± 0.420
8	Test condition 2 (plankton poised)	Yes	Yes	4.93 ± 0.430

Supplementary Table 8. ***Atp1*** **series gene expression reported as log_2 fold-change.**

Locus	Annotation	Acetate	*P*-value	Butyrate	*P*-value	H_2	*P*-value	*Iron	*P*-value	Poised electrode	*P*-value
Rpal_0170	ATP synthase F1, epsilon subunit	-1.37	0.387	0.900	0.957	2.87	0.094	3.86	0.009	3.59	0.003
Rpal_0171	ATP synthase F1, beta subunit	-0.317	0.901	0.156	0.560	2.04	0.340	3.29	0.068	3.71	0.008
Rpal_0172	ATP synthase F1, gamma subunit	-0.536	0.812	-2.06	0.481	2.49	0.249	3.86	0.027	3.19	0.011
Rpal_0173	ATP synthase F1, alpha subunit	-0.820	0.633	2.49	0.220	2.68	0.126	-3.42	0.024	-3.89	0.001
Rpal_0174	ATP synthase F1, delta subunit	0.020	0.993	4.01	0.009	0.873	0.846	2.75	0.095	-2.99	0.011
Rpal_0914	ATP synthase F0, A subunit	-0.207	0.940	2.42	0.332	1.98	0.408	3.53	0.044	-4.10	0.005
Rpal_0911	H^+-transporting two-sector ATPase B/B' subunit	-0.935	0.647	1.36	0.787	3.40	0.069	-4.40	0.010	5.06	0.001
Rpal_0913	H^+-transporting two-sector ATPase C subunit	-0.415	0.857	1.14	0.872	2.62	0.200	4.19	0.011	3.85	0.009
	**Average fold change**	**0.572**		**1.78**		**2.37**		**-3.66**		**3.80**	

All log_2 fold-change values are from a comparison of the conditions listed to aerobic chemoheterotrophic growth on yeast-extract peptone (see methods for further details).
Acetate = Photoheterotrophic growth using acetate
Butyrate = Photoheterotrophic growth using butyrate
Hydrogen = Photoautotrophic hydrogen oxidation
Iron = Photoautotrophic iron oxidation
Poised electrode = Extracellular electron uptake
* = Growth condition for which values are from n = 2 biological replicates, otherwise n = 3
P-values were determined in DESEQ2 (see Methods)
Reactions were determined from KEGG (https://www.kegg.jp) pathway analysis of the analyzed genes

Supplementary Table 9. *Atp2* series gene expression reported as log_2 fold-change.

Locus	Annotation	Acetate	*P*-value	Butyrate	*P*-value	H_2	*P*-value	*Iron	*P*-value	Poised electrode	*P*-value
Rpal_1049	H^+-transporting two-sector ATPase gamma subunit	-0.320	0.637	-1.02	0.787	-1.26	0.224	-0.648	0.379	1.60	0.734
Rpal_1050	ATP synthase F1, alpha subunit	-0.872	0.209	-1.33	0.507	-1.80	0.048	-0.823	0.363	0.916	0.646
Rpal_1051	H^+-transporting two-sector ATPase B/B' subunit	-1.06	0.284	-0.963	0.916	-1.77	0.196	-0.488	0.835	1.87	0.238
Rpal_1052	ATP synthase F0, C subunit	-0.488	0.771	-0.425	0.792	-1.34	0.564	-0.592	0.832	1.39	0.236
Rpal_1053	ATP synthase F0, A subunit	0.470	0.755	-1.21	0.793	-1.29	0.570	0.162	0.882	0.791	0.157
Rpal_1055	F0F1-ATPase subunit	0.579	0.828	-0.231	0.801	-0.317	0.924	0.908	0.715	4.71	0.001
Rpal_1056	alternate F1F0 ATPase, F1 subunit epsilon	1.13	0.241	0.854	0.050	-0.357	0.868	1.33	0.189	2.18	0.003
Rpal_1057	ATP synthase F1, beta subunit	1.10	0.641	-0.968	0.954	-0.507	0.989	1.53	0.517	4.52	0.006
	**Average fold change**	**0.069**		**-0.662**		**-1.08**		**0.172**		**2.25**	

All log_2 fold-change values are from a comparison of the conditions listed to aerobic chemoheterotrophic growth on yeast-extract peptone (see methods for further details).
Acetate = Photoheterotrophic growth using acetate
Butyrate = Photoheterotrophic growth using butyrate
Hydrogen = Photoautotrophic hydrogen oxidation
Iron = Photoautotrophic iron oxidation
Poised electrode = Extracellular electron uptake
* = Growth condition for which values are from n = 2 biological replicates, otherwise n = 3
P-values were determined in DESEQ2 (see Methods)
Reactions were determined from KEGG (https://www.kegg.jp) pathway analysis of the analyzed genes

Supplementary Table 10. Gas chromatography barrier ionization discharge (GC-BID) quantification of H_2 and CO_2 consumption. GC-BID analysis of TIE-1 wild-type (WT) and *ruBisCO* double mutant cells during photoautotrophic growth on H_2. Data are means ± s.e.m. of three biological replicates. Source data are provided as a Source Data File.

Strain	H_2 (μM)	CO_2 (μM)
WT	3957 ± 211.3	5395.5 ± 594.0
Δform I Δform II	717.0 ± 418.0	774.2 ± 8.90

Supplementary Table 11. Primers used in this study for plasmid construction.

Primer	Sequence
**Plasmid construction**	
Rpal_1747_upfor	CATATGGCGGCCGCCGCAGGTCCATTGCAGTCGT
Rpal_1747_uprev	CATATGGGATCCGTCGTCCTCCTTGAAAGCCCTGGC
Rpal_1748_dnfor	CATATGGGATCCTACGGAGGCTGATCGTGGAC
Rpal_1748_dnrev	CATATGCTGCAGGACCAAGACGAGCATCAGCGT
Rpal_5122dnfor	CATATGACTAGTTAAGCTGGCCTAGTCGACACG
Rpal_5122dnrev	CATATGGGATCCAGTGCACCGAGACCCGACAG
Rpal_5122uprev	CATATGACTAGTGGTGATCTCCTGCAATGCGAG
Rpal_5122upforNotI	CATATGGCGGCCGCGACATCATGCTGGCGAAGATGAT
cbbL-For	CATATGAACGAAGCAGTCACCAT
cbbLS-Rev	ACTAGTTCAGCCTCCGTAGC
pSRKGm-CbbM-For4-AclI-NdeI	AACGTTCATATGGACCAGTCGAACCGCTACG
pSRKGm-CbbM-Rev3-AclI-SmaI	CCCGGGTTACGCCGCCTGCG
**Plasmid Sanger sequencing**	
pSRKGM-For1-seq	TATGCTTCCGGCTCGTATGT
pSRKGM-CbbL-Rev1-Seq	GAACTTGTCCATGCGCTCGC
cbbLS-Rev1-seq	TGCAGCAGCTGATGCATCTG
Rpal_5122-PCR-screen_F	CAAAGAGAGCGAGCTGATCG
Rpal_5122 P6 Knockout Rev	TGATTACCGAGGACGCTGCTG

Supplementary Table 12. Primers used in this study for RT-qPCR.

Gene name (locus tag)	Primer name	Sequence
ruBisCO form I (Rpal_1747)	Rpal_1747qPCR_for	ACCAAGGACGACGAGAACAT
ruBisCO form I (Rpal_1747)	Rpal_1747qPCR_rev	CATGCAGTATTGGAAGCGCT
ruBisCO form II (Rpal_5122)	Rpal_5122qPCR_for	GGCGTATCTCAAGCTGTTCG
ruBisCO form II (Rpal_5122)	Rpal_5122qPCR_rev	CGATGAAGCCACCGTTGATC
clpX (Rpal_3308)	TIE-1clpXqRT-PCRFor	GGAGATCTGCAAGGTTCTCG
clpX (Rpal_3308)	TIE-1clpXqRT-PCRRev	CCGCTTGTAGTGATTGTGGA
recA (Rpal_4376)	TIE-1recAqRT-PCRFor	ATCGGCCAGATCAAGGAAC
recA (Rpal_4376)	TIE-1recAqRT-PCRRev	GAATTCGACCTGCTTGAACG
photosynthetic reaction center L subunit (Rpal_1716)	Rpal_1716_pufL_for	GAGAAGAAATACCGCGTTCG
photosynthetic reaction center L subunit (Rpal_1716)	Rpal_1716_pufL_rev	CCGAAGATCCCAACGTAGAA
F-type H+-transporting ATPase subunit beta (Rpal_1057)	Rpal_1057_atp1_for	ATTCTGAACGCCATCGAAAC
F-type H+-transporting ATPase subunit beta (Rpal_1057)	Rpal_1057_atp1_rev	GACGGTCGATTCACCAAGAT
F-type H+-transporting ATPase subunit beta (Rpal_0171)	Rpal_0171_atp2_for	GACGATCGCGGAGTATTTTC
F-type H+-transporting ATPase subunit beta (Rpal_0171)	Rpal_0171_atp2_rev	CAGGCTGGATAACTCGCTTC
pioA (Rpal_0817)	pioAqRTPCRfor	AAATTTCGACGACACCATCGA
pioA (Rpal_0817)	pioAqRTPCRrev	CTTGGCGGCGAGGATCT
hydrogenase large subunit (Rpal_1153)	Rpal_1153qPCRfor	GTGCAACTGCTGTCGATCAT
hydrogenase large subunit (Rpal_1153)	Rpal_1153qPCRrev	CCAGCACGTTGTCGAGAC

Supplementary References

1. Casadaban, M. J. & Cohen, S. N. Analysis of gene control signals by DNA fusion and cloning in *Escherichia coli*. *J. Mol. Biol.* **138**, 179-207 (1980).
2. Jiao, Y., Kappler, A., Croal, L. R. & Newman, D. K. Isolation and characterization of a genetically tractable photoautotrophic Fe(II)-oxidizing bacterium, *Rhodopseudomonas palustris* strain TIE-1. *Appl. Environ. Microbiol.* **71**, 4487-4496 (2005).
3. Khan, S. R., Gaines, J., Roop, R. M. & Farrand, S. K. Broad-host-range expression vectors with tightly regulated promoters and their use to examine the influence of TraR and TraM expression on Ti plasmid quorum sensing. *Appl. Environ. Microbiol.* **74**, 5053-5062 (2008).
4. Quandt, J. & Hynes, M. F. Versatile suicide vectors which allow direct selection for gene replacement in gram-negative bacteria. *Gene* **127**, 15-21 (1993).
