# Supplementary material for: Phototrophic extracellular electron uptake is linked to carbon dioxide fixation in the bacterium Rhodopseudomonas palustris
Source: Nat Commun. 2019 Mar 22;10:1355. doi: 10.1038/s41467-019-09377-6 (PMC6430793; doi:10.1038/s41467-019-09377-6)
Supplement: Supplementary file 2 — Reporting Summary [file 41467_2019_9377_MOESM2_ESM.pdf]

nature research

Corresponding author(s): Arpita Bose

Reporting Summary

Nature Research wishes to improve the reproducibility of the work that we publish. This form provides structure for consistency and transparency in reporting. For further information on Nature Research policies, see Authors & Referees and the Editorial Policy Checklist.

Statistical parameters

When statistical analyses are reported, confirm that the following items are present in the relevant location (e.g. figure legend, table legend, main text, or Methods section).

n/a	Confirmed	
☐	☒	The exact sample size (*n*) for each experimental group/condition, given as a discrete number and unit of measurement
☐	☒	An indication of whether measurements were taken from distinct samples or whether the same sample was measured repeatedly
☐	☒	The statistical test(s) used AND whether they are one- or two-sided<br>*Only common tests should be described solely by name; describe more complex techniques in the Methods section.*
☐	☒	A description of all covariates tested
☐	☒	A description of any assumptions or corrections, such as tests of normality and adjustment for multiple comparisons
☐	☒	A full description of the statistics including central tendency (e.g. means) or other basic estimates (e.g. regression coefficient) AND variation (e.g. standard deviation) or associated estimates of uncertainty (e.g. confidence intervals)
☐	☒	For null hypothesis testing, the test statistic (e.g. *F*, *t*, *r*) with confidence intervals, effect sizes, degrees of freedom and *P* value noted<br>*Give P values as exact values whenever suitable.*
☒	☐	For Bayesian analysis, information on the choice of priors and Markov chain Monte Carlo settings
☒	☐	For hierarchical and complex designs, identification of the appropriate level for tests and full reporting of outcomes
☒	☐	Estimates of effect sizes (e.g. Cohen's *d*, Pearson's *r*), indicating how they were calculated
☐	☒	Clearly defined error bars<br>*State explicitly what error bars represent (e.g. SD, SE, CI)*

Our web collection on statistics for biologists may be useful.

Software and code

Policy information about availability of computer code

Data collection	NIS Elements confocal imaging software (Nikon Corp.), Cameca WinImage software (Ametek Inc.), Bioconductor DESeq2 package (https://bioconductor.org/packages/release/bioc/html/DESeq2.html), Gen5 data analysis software (BioTek Instruments, Inc), Gamry Echem Analyst (Gamry Instruments Inc.), LabSolutions LC/GC Workstation (Shimadzu Corp.), Primer3 (http://primer3.ut.ee)
Data analysis	Fiji Image Processing package (https://fiji.sc), Excel for Mac (Microsoft Corp.), RStudio (https://www.rstudio.com), Origin Graphing and Analysis software (OriginLab Corp.), Trimmomatic version 0.36, TopHat2 version 2.1.1, Bowtie 2 version 2.3.3.1, HTSeq version 0.9.1, DESEQ2 version 1.16.1, ggplot2

For manuscripts utilizing custom algorithms or software that are central to the research but not yet described in published literature, software must be made available to editors/reviewers upon request. We strongly encourage code deposition in a community repository (e.g. GitHub). See the Nature Research guidelines for submitting code & software for further information.

April 2018

Data

Policy information about availability of data

All manuscripts must include a data availability statement. This statement should provide the following information, where applicable:

- Accession codes, unique identifiers, or web links for publicly available datasets
- A list of figures that have associated raw data
- A description of any restrictions on data availability

All data in this study are available from the corresponding authors upon request. The source data underlying Figs 1d, 2a-b, 3a-d, 4a-c, 5a-i, 6a-c; Supplementary Figs 2a-d, 3, 4, 5d-e, 6a-i, 7a-b, 8-11; and Supplementary Tables 1-3 and 5-7 and 10 are provided as a Source Data file. Sequencing reads used for differential expression analysis are available under BioProject PRJNA417278.

Field-specific reporting

Please select the best fit for your research. If you are not sure, read the appropriate sections before making your selection.

☒ Life sciences ☐ Behavioural & social sciences ☐ Ecological, evolutionary & environmental sciences

For a reference copy of the document with all sections, see nature.com/authors/policies/ReportingSummary-flat.pdf

Life sciences study design

All studies must disclose on these points even when the disclosure is negative.

Sample size	No sample size calculations were performed to predetermine sample size. The experiments were performed at least three times independently to allow for statistical inference. The standard error was determined from those data.
Data exclusions	Only data from failed experiments, due to technical errors or microbiological contamination, were excluded.
Replication	Replicate experiments were successful. Where applicable, sample size (n) and the number of technical replicates are indicated in the figure legends or the main text.
Randomization	We believe this not applicable in our case.
Blinding	We believe this not applicable in our case.

Reporting for specific materials, systems and methods

Materials & experimental systems

n/a	Involved in the study	
☐	☒	Unique biological materials
☒	☐	Antibodies
☒	☐	Eukaryotic cell lines
☒	☐	Palaeontology
☒	☐	Animals and other organisms
☒	☐	Human research participants

Methods

n/a	Involved in the study	
☒	☐	ChIP-seq
☒	☐	Flow cytometry
☒	☐	MRI-based neuroimaging

Unique biological materials

Policy information about availability of materials

Obtaining unique materials	The bacterial strains and plasmids generated and/or used in this study in this study are available from the corresponding author upon request.
